# Roundup causes embryonic development failure and alters metabolic pathways and gut microbiota functionality in non-target species

**DOI:** 10.1186/s40168-020-00943-5

**Published:** 2020-12-15

**Authors:** Antonio Suppa, Jouni Kvist, Xiaojing Li, Vignesh Dhandapani, Hanan Almulla, Antoine Y. Tian, Stephen Kissane, Jiarui Zhou, Alessio Perotti, Hayley Mangelson, Kyle Langford, Valeria Rossi, James B. Brown, Luisa Orsini

**Affiliations:** 1 Environmental Genomics Group, School of Biosciences, the University of Birmingham, Birmingham, B15 2TT UK; 2 Department of Chemistry, Life Sciences and Environmental Sustainability University of Parma, Department of Life Sciences, Viale Usberti, 11/A, Parma, Italy; 3 School of Biosciences, University of Birmingham, Birmingham, B15 2TT UK; 4 Computer Science, University of Birmingham, Birmingham, UK; 5 Phase Genomics, Seattle, WA USA; 6 Environmental Bioinformatics, Centre for Computational Biology, School of Biosciences, University of Birmingham Edgbaston, Birmingham, B15 2TT UK; 7 Environmental Genomics and Systems Biology Division, Lawrence Berkeley National Laboratory, Berkeley, CA 94720 USA; 8 Statistics Department, University of California, Berkeley, Berkeley, CA, 94720 USA, Preminon LLC, Rodeo, CA 94572 USA

## Abstract

**Background:**

Research around the weedkiller Roundup is among the most contentious of the twenty-first century. Scientists have provided inconclusive evidence that the weedkiller causes cancer and other life-threatening diseases, while industry-paid research reports that the weedkiller has no adverse effect on humans or animals. Much of the controversial evidence on Roundup is rooted in the approach used to determine safe use of chemicals, defined by outdated toxicity tests. We apply a system biology approach to the biomedical and ecological model species *Daphnia* to quantify the impact of glyphosate and of its commercial formula, Roundup, on fitness, genome-wide transcription and gut microbiota, taking full advantage of clonal reproduction in *Daphnia.* We then apply machine learning-based statistical analysis to identify and prioritize correlations between genome-wide transcriptional and microbiota changes.

**Results:**

We demonstrate that chronic exposure to ecologically relevant concentrations of glyphosate and Roundup at the approved regulatory threshold for drinking water in the US induce embryonic developmental failure, induce significant DNA damage (genotoxicity), and interfere with signaling. Furthermore, chronic exposure to the weedkiller alters the gut microbiota functionality and composition interfering with carbon and fat metabolism, as well as homeostasis. Using the “Reactome,” we identify conserved pathways across the Tree of Life, which are potential targets for Roundup in other species, including liver metabolism, inflammation pathways, and collagen degradation, responsible for the repair of wounds and tissue remodeling.

**Conclusions:**

Our results show that chronic exposure to concentrations of Roundup and glyphosate at the approved regulatory threshold for drinking water causes embryonic development failure and alteration of key metabolic functions via direct effect on the host molecular processes and indirect effect on the gut microbiota. The ecological model species *Daphnia* occupies a central position in the food web of aquatic ecosystems, being the preferred food of small vertebrates and invertebrates as well as a grazer of algae and bacteria. The impact of the weedkiller on this keystone species has cascading effects on aquatic food webs, affecting their ability to deliver critical ecosystem services.

Video Abstract

**Supplementary Information:**

The online version contains supplementary material available at 10.1186/s40168-020-00943-5.

## Background

The weedkiller Roundup (Bayer), first developed by Monsanto in the 1970s, is to date the most used non-selective herbicide by volume (6 billion kg are applied worldwide) [1]. The research around Roundup is highly contentious: some scientists have claimed that it causes pathologies ranging from cancer (e.g. [2]) to celiac disease [3] and autism [4], while industry-paid research reports that the herbicide has no untoward effects. Much of the controversial evidence on Roundup is rooted in outdated toxicity tests, called LC50 [5], from which safe use of chemicals in the environment is extrapolated. These outdated tests are the current state-of-the-art in regulatory science [6]. To allow risk managers to make informed decisions, new methodologies that include ecologically relevant concentrations of chemicals and their mixtures, analysis of the long-term impact of chemicals on multiple species and models to predict adverse effects are needed (e.g. [7]). The adverse effect of Roundup documented to date is based on unrealistic concentrations of the compound; it has been largely correlative and missing, by design, potential pathological effects that may arise from long-term exposures to sublethal doses [1].

Roundup and its active ingredient glyphosate are expected to be innocuous to animals because they target an enzyme only found in plants and microorganisms (EPSPS [8]). However, animals rely on a specialized gut microbiota for growth, immunity, and pathogen defense [9]. Growing evidence shows that the weedkiller has a proven indirect adverse effect on vertebrates and invertebrates via the gut microbiota (e.g. [10–15]).

In the past, Roundup was not considered a problem for ground and surface waters [16]. It was later discovered that agricultural and urban run-off are responsible for the weedkiller leaching into surface water. Dissolved glyphosate (or its metabolite AMPA) sorbs to the sediment of water bodies [17], extending its half-life to 130 days and becoming persistent in water reservoirs worldwide [17, 18]. Because of its enhanced persistence in the environment, glyphosate is found in sewage and stormwater overflows [19], in outlets from wastewater treatment plants [18], and in drinking water [20]. Reports of glyphosate, Roundup, and their metabolite AMPA in surface and drinking water vary greatly with the geographic area and are generally more severe in the USA (e.g. [21]). Concentration of these compounds in the environment varies depending on whether the parent products alone or the parent products and the metabolite AMPA are concurrently quantified [17]. An understanding of the functional pathways modulated by ecologically relevant doses of the weedkiller, both at organismal level and on the gut microbial community, is needed to resolve the ongoing debate on Roundup.

Here, we apply a system biology approach and quantify the fitness burden, the genotoxic effect, the transcriptional and gut microbiota changes of ecologically relevant concentrations of Roundup and glyphosate on the biomedical and ecological model species *Daphnia*. We then apply machine learning-based statistical analysis to identify and prioritize correlations between genome-wide transcriptional and microbiota changes. As the biomedical model species *Drosophila*, *Daphnia* shares a large proportion of its genes with other species, including vertebrates [22]. Using the functional analysis of protein domains, we identify conserved pathways across the Tree of Life modulated by Roundup and glyphosate, identifying pathways conserved across species that are altered by Roundup. *Daphnia* is common to standing freshwater habitats worldwide, where it is central to the aquatic foodweb functionality [23, 24]. Understanding the impact of the widely used weedkiller on this species informs us on its cascading effect on aquatic foodwebs and on the disruption of critical services delivered by freshwater ecosystems.

## Results

**Fig. 1:**
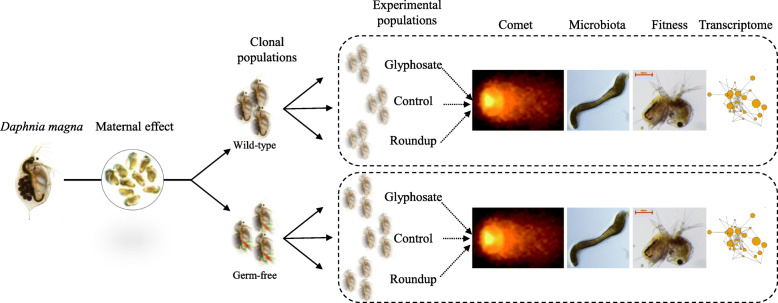
Experimental design. *Daphnia magna* isoclonal lines were obtained from four distinct genotypes; three of these genotypes were revived from dormant embryos LRV3.5_15, LRV13.5_1, and LRV13.2, whereas P-IT is a reference laboratory genotype. For each genotype, germ-free and wild-type clonal populations were obtained. Germ-free clonal populations were obtained by exposing wild-type clonal replicates of the four genotypes to a cocktail of antibiotics (20 mg/L of tetracycline, streptomycin, and ampicillin). Both wild-type and germ-free lines were exposed in triplicated to glyphosate, Roundup, and control conditions (non-stress) after at least two generations in standard laboratory conditions to reduce interference from maternal effect. For each biological replicate, genotype, and treatment, genotoxicity via the comet assay, gut microbiota composition, fitness, and whole-genome transcriptional profiles were measured.

Like *Drosophila*, *Daphnia* enjoys many technical advantages over vertebrate models: they are easy and inexpensive to culture in the laboratory, have a short life cycle, and produce large numbers of externally laid embryos. In addition, *Daphnia* has a parthenogenetic life cycle, in which sexual and asexual reproduction alternate. Sexual recombination results in early-stage embryos that arrest their development and enter dormancy [25]. Revived dormant embryos are genetically distinct and can be propagated indefinitely in the laboratory via clonal reproduction, allowing the rearing of populations of isogenic individuals (clones) from a single genotype [26, 27]. Capitalizing on these properties, we performed multiomics and fitness analyses on replicated clones of distinct *Daphnia* genotypes (Fig. 1). We used three genotypes hatched from dormant *Daphnia* embryos revived from Lake Ring, Denmark: LRV3.5_15, LRV13.5_1 and LRV13_2 [26, 28, 29]. We also used a laboratory reference strain, P-IT, provided by the Institute of Ecosystem study, CNR Verbania, Italy.

### Fitness burden of Roundup and glyphosate

We quantified the fitness burden resulting from the chronic exposure to ecologically relevant concentrations of Roundup and glyphosate, corresponding to the drinking water Maximum Contaminant Level (MCL) set by the U.S. Environmental Protection Agency of 1 mg/L for long-term exposures [30] (Dryad entry doi:10.5061/dryad.mcvdncjws). We found that these perturbations affected all fitness-linked life history traits (Table 1; Gly, Rou; Figure S1), except for mortality (Figure S2). The severity of the effect varied by genotype, suggesting that the genetic background affects fitness response (Table 1; G). Both compounds significantly reduced fecundity, increased developmental failure (measured as the number of aborted eggs and juveniles dead at birth), delayed maturation, and decreased size at maturity (Table 1; Figure S1). Exposures to both glyphosate and Roundup resulted in significant genotoxicity caused by DNA damage quantified with the comet assay [31], the severity of which was genotype-dependent (Table 1, G; Figure S3).

**Table 1:**
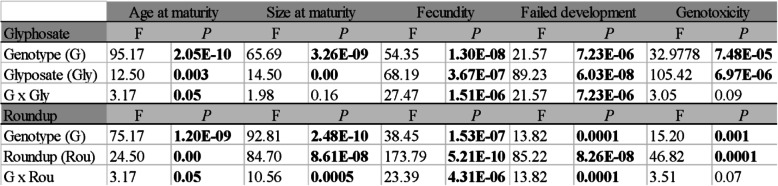
Analysis of variance. ANOVAs per individual fitness-linked life history traits (size and age at maturity, fecundity, and failed development) calculated for glyphosate and Roundup chronic exposures testing for the effect of genotype, treatment, and their interaction term. The genotoxic response to glyphosate and Roundup is also shown. Significant *P* values are shown in bold. Supporting figures are Figures S1 and S3

### Host transcriptome response to Roundup and glyphosate

**Fig. 2:**
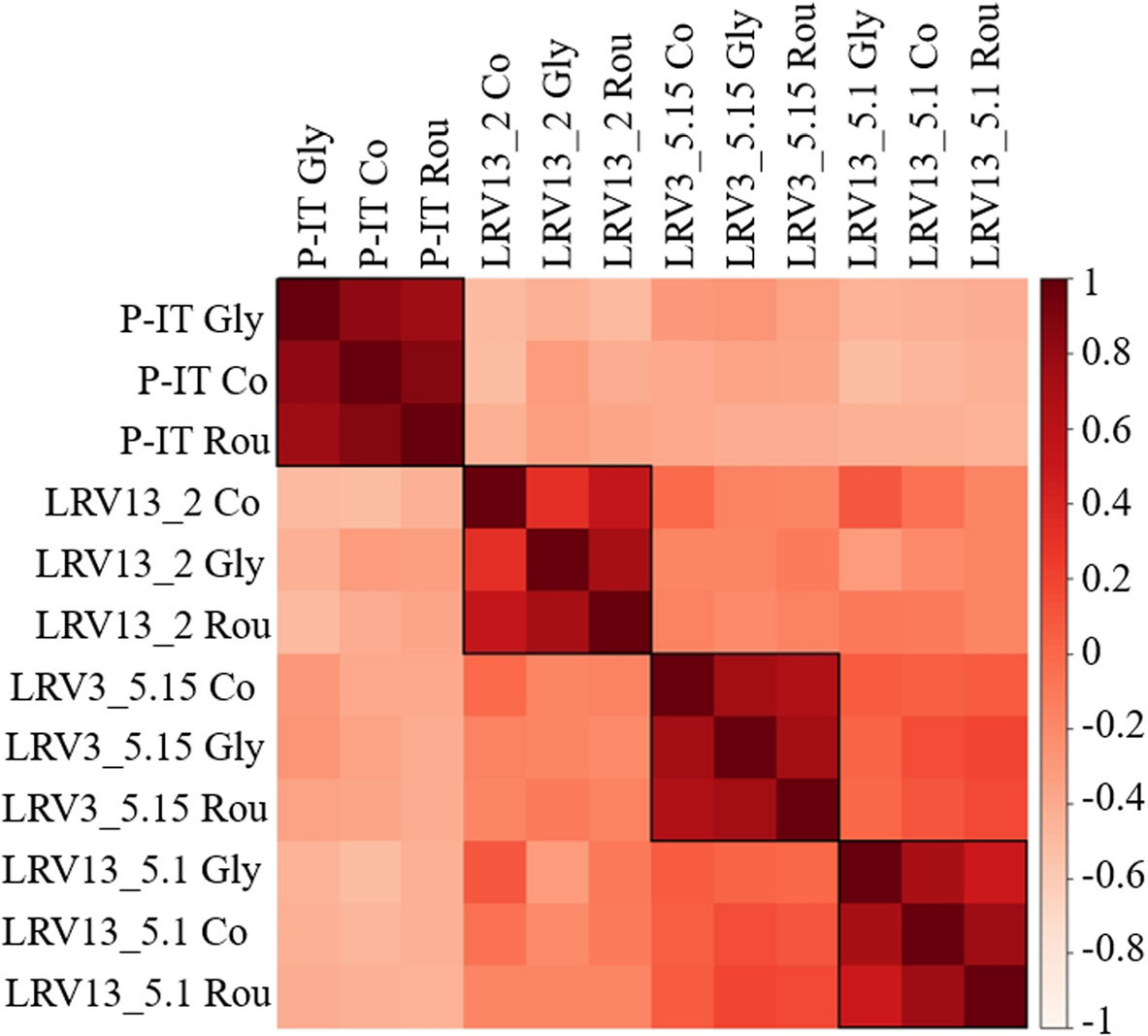
Genome-wide differential expression analysis. Heat maps of the genome-wide differentially expressed transcript (DEseq Padj < 0.01) among the four genotypes, measured with Pearson correlation: LRV3.5_15, LRV13.5_1, LRV13.2, and P-IT. The data are obtained from three biological replicates per genotype. Similarity increases from cream to dark red, as indicated by the color bar. The abbreviations for the environmental perturbations are as follows: Rou—Roundup; Gly—glyphosate; Co—control

We quantified the genome-wide transcriptional response of *Daphnia* to glyphosate and its commercial formula Roundup in replicated clones of the same four genotypes used in the fitness and genotoxic analysis both for control and exposed biological replicates (Fig. 1) (NCBI Bioproject PRJNA606209). Gene-level differential analysis between control (non-exposed) animals and animals exposed to glyphosate and Roundup did not identify any significant differentially expressed gene. These findings were supported by a genome-wide transcription profile analysis showing that individual treatment effects were obscured by genotype effects (Fig. 2; Figure S4).

To uncover the molecular machinery that underpins the severe fitness burden observed in the fitness traits and the genotoxic response, we performed a weighted gene co-expression network analysis, which enabled us to identify networks of genes that were modulated synchronously, even when their component genes were below the DE statistical thresholds [32]. These gene networks were then interrogated to identify enriched functional pathways. We discovered that approximately two thirds of the *Daphnia magna* genes, enriched for embryonic development (SCW, DPP), growth, morphogenesis (BMP), and basic metabolism (GABA, triacylglycerol, pyruvate), drive transcriptional early response and are shared among genetic backgrounds and treatments (Table S1; Module 14). This early stress response is followed by a condition-specific transcriptional response, resulting in a single module associated with glyphosate (Module 8) and four modules associated with Roundup (Modules 20, 23, 26, and 34; Table S2). Glyphosate-specific Module 8 was enriched (*P* < 0.05) for key metabolic pathways [cellular (heme); fatty acids (Butanoate); alanine, aspartate and glutamate metabolism], signaling pathways, cellular activities (e.g., ECM receptor), and Huntington disease pathway (Table S2). Genes enriched in these pathways include membrane transporters, transcription regulation, and protein-binding genes (Table S3). The functional domains of these genes searched across databases (Panther, Pfam Gene3D, etc.) include vitellogenin, hemolymph juvenile hormone (JHBP), protein transporters (Nonaspanin; immunoglobulin-like fold), redox mechanisms (e.g., aldehyde dehydrogenase), and hemostasis (von Willebrand factor, cytochrome b5, protoporphyrinogen oxidase) (Table S1). To identify conserved domains across the Tree of Life and potential targets of glyphosate and Roundup in other species, we used the “Reactome” [33]. Conserved gene domains, which may be of potential concern as targets for glyphosate in other species, include three main categories: liver metabolism (lipids and glucose), inflammation pathways (leukocytes), and collagen degradation, responsible for the repair of wounds and tissue remodeling (Table S1; Reactome).

Four co-expression gene modules, containing between 31 and 33 genes each (Modules 20, 23, 26, 34), were associated with Roundup (Table S2). Pathways enriched in these modules include RNA transport (Module 20) and fatty acid metabolism (Module 34) (Table S3). A domain analysis of these modules identified regulation of basic cellular function and trafficking: cellular DNA-binding, cell proliferation, membrane transport, methyl transferase, DNA repair (Endonuclease), sugar metabolism, and Proteolysis. The “Reactome” analysis revealed that very few pathways linked to Roundup were conserved between *Daphnia* and other species. The conserved domains across the Tree of Life regulated fundamental protein functions and membrane transport as well as cholesterol and sugar metabolism (Table S1; Reactome).

### Gut microbiota changes in response to glyphosate and Roundup

We quantified the impact of Roundup and glyphosate on the dynamics and composition of the gut microbiota. Replicated clones of the same four *D. magna* genotypes used in the fitness and transcriptome analysis were used (Fig. 1).

Firstly, we performed experiments to characterize the microbiota, establish its origin and its overall dynamics from gut colonization (48 h after birth) to last instar (144 h after birth) in response to different xenobionts; we then quantified the impact of Roundup and glyphosate on established gut communities. The reference microbiome, as well as the experiments investigating the dynamics and origin of the *Daphnia* gut microbiota, is in Additional file 1.

Having established that the *D. magna* gut microbiota stabilizes at the last instar (> day 4) and is strongly genetically determined (Additional file 1), we quantified the impact of glyphosate and Roundup on established gut communities (day 5). Having observed that establishment success of gut communities may be more severely affected when glyphosate and Roundup co-occur with antimicrobial agents (Additional file 1), we quantified the effect of both compounds on clonal replicates treated with antibiotics (germ-free) as well as on non-treated replicates (wild-type) (Fig. 1).

**Fig. 3:**
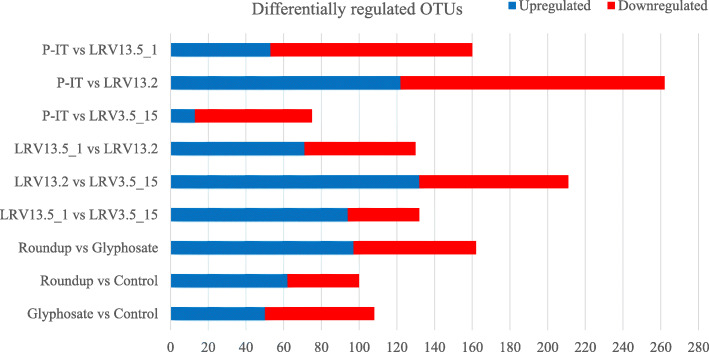
Differential sOTU abundance. Number of up- and downregulated sOTUs between treatments and pairwise genotypes. Differential analysis was done with DESeq2 using Padj < 0.05

**Fig. 4:**
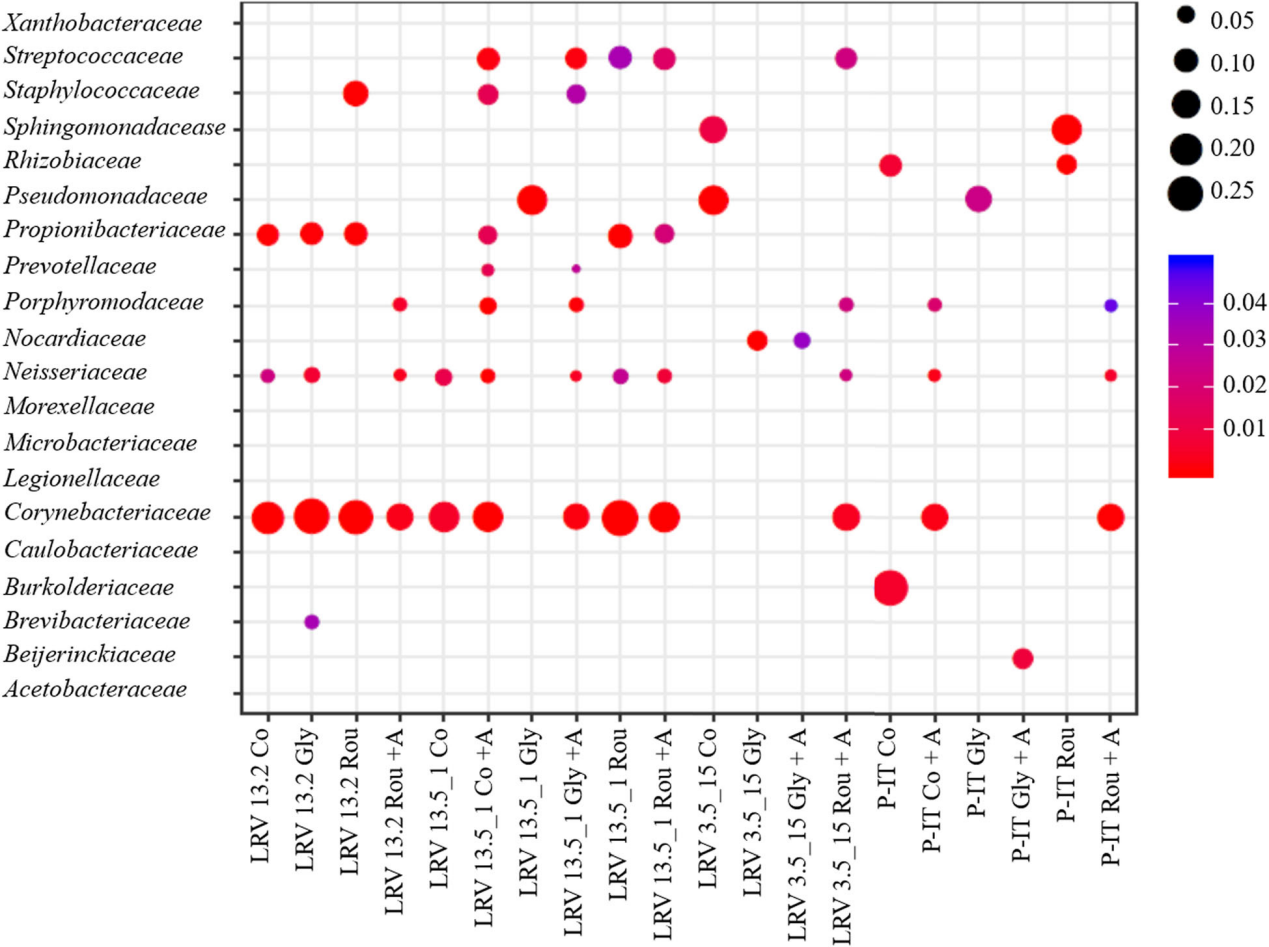
Enriched sOTUs. Enriched sOTU families within genotypes, across biological replicates, and conditions relative to the overall sOTU distribution. The relative abundance of each taxon is expressed by the size of the circle, whereas colors ranging from blue to red indicate significance levels. Co—control; Co+A—control with antibiotic treatment; Gly—glyphosate; Gly+A—glyphosate with antibiotic treatment; Rou—Roundup; Rou+A—Roundup with antibiotic treatment

**Fig. 5:**
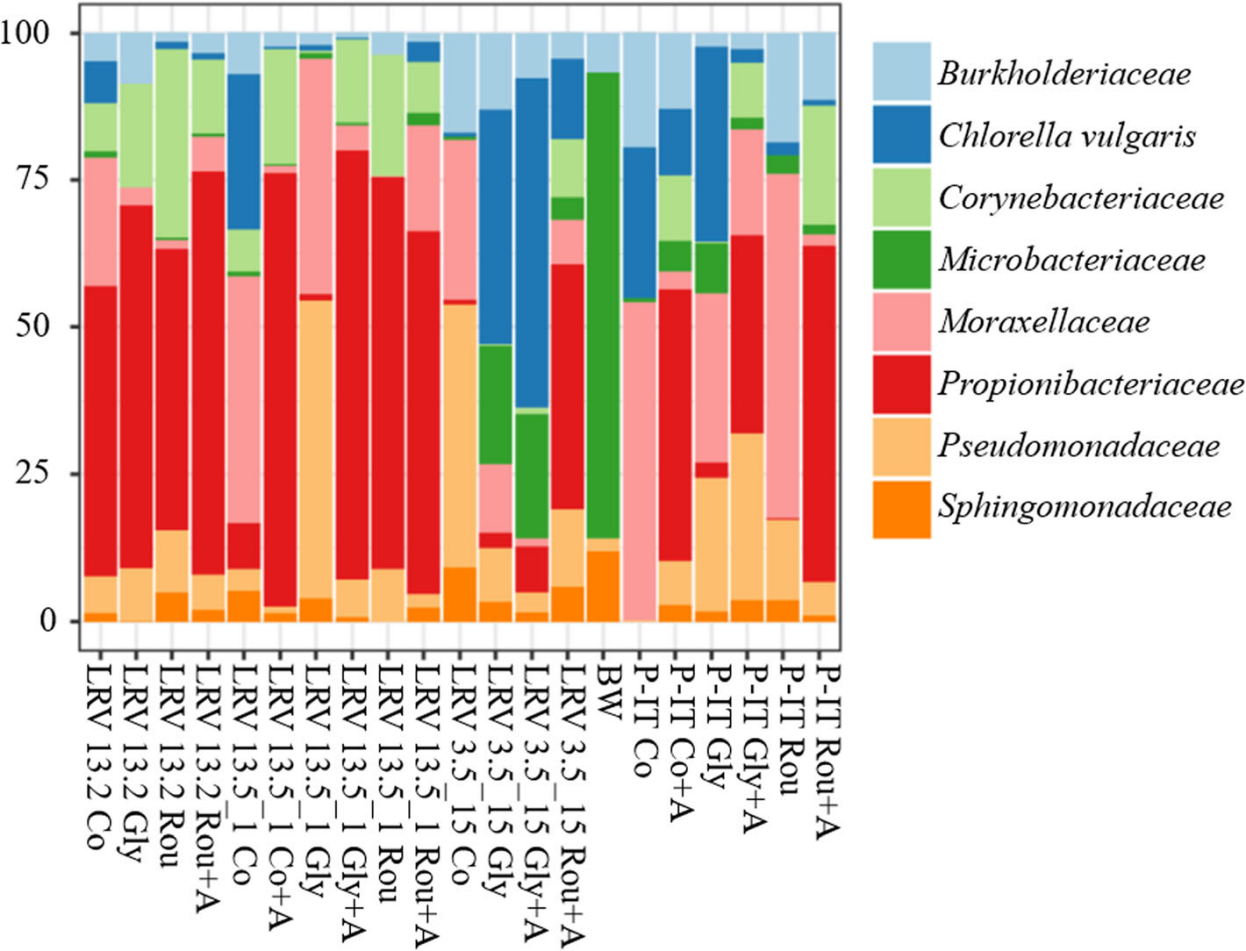
Microbiota composition after exposure to glyphosate and Roundup. Microbiota composition of established gut communities after exposure to Roundup and glyphosate. The top eight bacteria families are shown for the four genotypes across biological replicates. The bacterial composition of the borehole water (BW, non-sterile medium across 3 replicates), in which the exposures were conducted, is also shown. Listed treatments are as in Fig. 4. Barplot composition is missing for some antibiotic treatments because the genotypes, including all biological replicates, did not survive. The PERMANOVA statistics in Table 2 support this plot

Firstly, we confirmed that the composition of the gut microbiota is strongly genetically influenced by quantifying sOTUs exhibiting differential abundances between pairs of genotypes; they ranged between 262 (P-IT/LRV13.2) and 75 (P-IT/LRV3.5_15) (Fig. 3). Significant differences between pairs of genotypes were explained by different gut community composition (βετα diversity), as shown by the Jaccard and the Bray-Curtis similarity index analysis (Table S4). Secondly, we quantified the impact of glyphosate and Roundup on established communities. We observed that both glyphosate and Roundup significantly changed the relative abundance and composition of the established gut microbiota in a genotype-specific manner, as significant “genotype” and “genotype × treatment” terms indicate in the PERMANOVA analysis (Table 2; G; G × T). This response was driven by significant changes in the relative abundance of six Families (*Moraxellaceae*, *Burkholderiaceae*, *Beijerinckiaceae*, *Rhizobiaceae*, *Nocardiaceae*, *Flavobacteriaceae*) and seven genera (*Acinetobacter*, *Acidovorax*, *Limnohabitans*, *Legionella*, *Galbitalea*, *Rhodococcus*, *Flavobacterium*) (Fig. 4), which are all non-core taxa (Table S5). Glyphosate and Roundup affected gut microbiota dynamics in established gut communities in a genotype-specific manner (Table 2, G × T), leading to different gut microbiota composition among genotypes (Fig. 5; Table 2, G). Prior treatment with antibiotics significantly affected gut recolonization in a genotype-specific manner as the significant “genotype × antibiotic treatment” and the three-way interaction terms in the PERMANOVA analysis shows (Table 2, G × A; G × T × A). A hierarchical clustering of sOTUs including all treatments and samples confirmed that the sample variance was firstly explained by the antibiotic treatment (33% of the variance), except for genotype LRV 13_2, for which the antibiotic treatment did not work as well as in other genotypes likely due to experimental error (Figure S5A), and then by the genotype (Figure S5B).

**Table 2:** PERMANOVA. Permutational Multivariate Analysis of Variance using Bray-Curtis [34] distance sOTU matrices testing for the effect of genotype (G), treatment (Roundup and glyphosate) (T), antibiotic treatment (A), and their interaction terms. Significant terms are in bold

	Df	R^2^	*P*
Genotype (G)	3	0.19	**1E−04**
Treatment (T)	2	0.05	0.07
Antibiotic (A)	1	0.14	**1E−04**
G × T	6	0.13	**0.03**
G × A	3	0.07	**0.03**
T × A	2	0.03	0.26
G × T × A	2	0.07	**0.006**

The bacterial families of established gut communities affected by glyphosate and Roundup (Fig. 5) do not overlap with the bacteria families perturbed during gut colonization (Fig. A4C in Additional file 1).

### Correlations between transcriptome and microbiome changes

The host transcriptome analysis identified non-significant signatures at individual gene level, whereas it clearly identified gene networks and pathways impacted by Roundup and glyphosate. Traditional statistical methods, which are asymptotic in nature, are not suitable to identify correlative changes between the multidimentional transcriptome and microbiome datasets. Therefore, we applied machine learning-based statistical analysis to identify correlations between genome-wide transcriptional changes and microbiota compositional changes induced by glyphosate and Roundup chronic exposures, using the Random Forest classifier and the Random Forest regression [35]. We then performed a functional analysis of the transcriptional co-expression networks significantly correlated to changes in microbiota composition to identify enriched pathways conserved across the Tree of Life.

Glyphosate-induced host transcriptional changes were enriched for a neuroactive ligand receptor, the Fanconi anemia (FA) pathway, and DNA repair [36], both significantly associated with gut community alteration (Table S6). The three glyphosate-enriched pathways significantly correlated with 9 bacteria sOTUs including *Dietziaceae*, *Nocardiaceae*, *Microbacteriaceae*, *Actinobacteria*, *Streptococcaceae*, *Caulobacteraceae*, *Sphingomonadaceae*, *Burkholderiaceae*, and *Moraxellaceae* (Table S6)*.* Of these, four sOTU families (39%) are core taxa and four are part of the same phylum (*Actinobacteria*).

Roundup-induced host transcriptional changes enriched for lipid metabolism and signaling pathways were significantly correlated with microbiota shifts. The Roundup-enriched pathways conserved across the Tree of Life were as follows: the cannabinoid, the Wtn, the Hedgehog (Hh), the Forkhead box protein O (FoxO), and the transforming growth factor (TGF-beta) pathways (Table S6). These pathways were significantly correlated with the gut bacteria phylum *Actinobacteria* and 6 sOTU families: *Micrococcaceae*, *Flavobacteriaceae*, *Staphylococcaceae*, *Saccharimonadales*, *Rhizobiaceae*, *Sphingomonadaceae*, *Burkholderiaceae* and *Betaproteobacteriaceae* (Table S6). Of these sOTUs, 15% belongs to core taxa (Table S6). Roundup-linked pathways are functionally associated with changes in *Actinobacteria*. Shifts in non-core taxa induced by glyphosate and Roundup resulted in significant alterations of key functional pathways (Table S7). Both glyphosate and Roundup overwhelmingly enriched carbon metabolism, and then lipid metabolism, signal transduction, and detoxification pathways (Table S7).

## Discussion

We found that that chronic exposure to glyphosate and its commercial formula, Roundup, corresponding to the US drinking water Maximum Contaminant Level (MCL) [30] have severe adverse effects on the non-target aquatic invertebrate *Daphnia*, inducing embryonic developmental failure and genotoxic effect. The fitness burden imposed by exposure to sublethal concentrations of Roundup and glyphosate has been shown in other non-target species (e.g., medaka fry [37]; zebrafish [38]). The observed effects range from low hatching success to embryo mortalities and developmental abnormalities. Evidence of genotoxicity has been shown in other species, including human cell lines [39] (tadpoles [40], caimans [41], fish [42, 43], lizards [44], shrimps [45], and invertebrates [46]), even though genotoxicity on human cell lines is controversial [47].

We observed that the molecular machinery underpinning the phenotype induced by glyphosate and Roundup exposures was not explained by individual gene regulation. Similar lack of individual gene signature has been previously observed in *Daphnia* exposed to sublethal environmental perturbations [32], whereas lethal concentrations of xenobionts have been shown to induce significant differential modulation of individual genes [48–51]. To gain an understanding of the molecular machinery underpinning the fitness burden and genotoxic effect imposed by Roundup and glyphosate, we investigated pathways enriched by exposure to glyphosate and Roundup. This analysis revealed functional pathways regulating metabolism, immunity, oxidative stress, cell division, and hormone regulation to be significantly affected. Our findings are consistent with previous studies associating inflammation, cell proliferation, apoptosis, and immunity to glyphosate exposures in non-target species (e.g., brown trout [52]; rats [53]). Genes underpinning stress response, collagen homologues, lipid metabolism, and vitellogenin which were enriched in our experimental animals within these pathways have been previously linked to glyphosate in other invertebrates (e.g., springtail [54]). Disruption of lipid and sugar metabolism by the herbicide Roundup has also been shown in vertebrates (e.g., rats) for which kidney and liver tissue damage, as well as gene expression consistent with fibrosis and necrosis, were observed [53]. One of the gene networks significantly associated with glyphosate exposure was histone modification, a known methylation mechanism, suggesting that epigenetic mechanisms play a role in *Daphnia* response to glyphosate.

We found that early exposure to the xenobiotic agents has a strong impact on the dynamics, establishment, and function of the microbiome, which has been observed in other species (e.g., mice) [55]. Our results also indicate that glyphosate and Roundup had a significant impact on low-abundant gut bacteria taxa, which were severely affected in the established communities, whereas core bacterial taxa were not significantly affected. This pattern is explained by core microbes maintained through low competition and synergistic interactions, as well as intraspecies strain variability, which makes them less susceptible than non-core taxa [56]. However, low-abundant taxa are main drivers of community composition and, hence, important to the function of the microbiota [57]. Indeed, shifts in these non-core taxa caused by glyphosate and Roundup exposure contributed to significant alterations of key functional pathways. Specifically, carbon and lipid metabolism, signal transduction, and detoxification pathways were overwhelmingly enriched following exposure to Roundup and glyphosate. Previous studies in bacteria support that glyphosate affects both carbon and fat metabolic pathways [58]. In our experiment, *Flavobacteria* and *Rhizobiaceae*, whose relative abundance changed significantly in presence of glyphosate and Roundup, are bacteria found commonly in activated sludge or soil enriched with glyphosate; these bacteria are able to use glyphosate or other organophosphate compounds as the sole source of carbon after cleaving the C–P bond [58–60]. The significant shifts in these bacteria observed in the experimental exposures indicate that the weedkiller significantly alters the gut microbiota within a single generation at doses well below the accepted toxicity regulatory thresholds.

We found that functional host pathways enriched by exposure to glyphosate and Roundup were associated with significant alterations of the gut microbiota both in composition and function. The glyphosate-induced host transcriptional changes enriched for a neuroactive ligand-receptor, the Fanconi anemia (FA) pathway, and DNA repair [36] were significantly associated with 9 bacteria families of the genus *Actinobacteria*. The Roundup-induced host transcriptional changes including the cannabinoid, the Wtn, the Hedgehog (Hh), the Forkhead box protein O (FoxO), and the transforming growth factor (TGF-beta) pathways were also significantly associated with changes in *Actinobacteria*. This family of bacteria is abundant in a wide range of aquatic and terrestrial environments and also forms an important component of higher organism microbiota, including plants [61]. *Actinobacteria* also play a key role in human gut homeostasis [62]. The perturbation (dysbiosis) of gut microbiota linked to *Actinobacteria* has been associated not only with intestinal disorders but also with numerous extra-intestinal diseases such as metabolic and neurological disorders [63]. This bacterial group is so important that probiotic supplements containing *Actinobacteria* are used to improve gut microbiota health in the prevention of degenerative diseases, such as obesity, diabetes, cancer, cardiovascular diseases, liver disease, and inflammatory bowel disease [64]

The functional pathways which dynamics correlates with gut bacteria alteration were conserved across species, showing orthology across the Tree of Life. The neuroactive ligand receptor pathway was orthologous with a number of endocrine and disease pathways, including hormone dysfunctions [65] and metabolic disorders [66]. The Fanconi anemia (FA) pathway has been implicated in the sensitivity of cancer cells to DNA crosslinking agents [36]. Also orthologous to other species pathways were as follows: (i) the Wtn pathway, which regulates cell fate, migration, and organogenesis during embryogenesis (e.g. [67]); (ii) the Hh pathway, which regulates differentiation, proliferation, and stem cell population. The Hh pathway was first discovered for regulating cuticle formation in *Drosophila* [68]. The core components of the Hh pathway initially identified in *Drosophila* are conserved in vertebrates, where the pathway has maintained the same mechanisms of action [69]. This pathway has been associated with many types of cancer, including skin, leukemia, lung, brain, and gastrointestinal cancers [69]; (iii) FoxO pathway, which regulates a variety of cellular processes including apoptosis, proliferation, cell cycle progression, DNA damage, and tumorigenesis. It also responds to several cellular stresses such as UV irradiation and oxidative stress [70], and (iv) TGF factor, which regulates cell developmental programs and behavior, such as proliferation, differentiation, morphogenesis, tissue homeostasis, and regeneration [71]. Conservation of function and identical biological outcomes have been shown only for the Hh pathway. For the other pathways, ad hoc experiments are needed to assess conservation of function. However, our results supported by multiple lines of evidence and previous findings suggest that these same pathways may be perturbed in other organisms exposed to glyphosate and Roundup.

## Conclusions

Overall, our results show that chronic exposure to concentrations of Roundup and glyphosate well below the approved regulatory threshold causes embryonic developmental failure and alteration of metabolic pathways via direct effect on the host and indirect effect on the gut microbiota in the keystone grazer *Daphnia*. As *Daphnia* is central to the food web of aquatic ecosystems and an indicator of ecosystem health, the weedkiller can potentially impose a fitness burden on freshwater aquatic foodwebs, affecting their ability to deliver critical ecosystem services (e.g., clean water, nutrient cycling). The conservation of many of the perturbed pathways identified in *Daphnia* across the Tree of Life calls for a thorough assessment of chronic exposure to the weedkiller at sublethal concentrations in non-target species, including humans.

## Methods

The impact of glyphosate and of its commercial formula Roundup (Bayer) were quantified on *D. magna* by measuring changes in fitness-linked life history traits, genome-wide transcription, and the microbiota. Four genotypes were used: LRV3.5_15, LRV13.5_1 and LRV13_2 were previously isolated from a shallow lake in Denmark [26, 28, 29]; P-IT is a laboratory reference strain provided by the Institute of Ecosystem study, CNR Verbania, Italy. Host response was measured in multiple fitness-linked life history traits and at genome-wide transcriptional level. Gut microbiome response was quantified within established gut communities (day 5) in a split design in which replicated clones of the same genotypes were either directly exposed to the treatments (wild-type) or exposed after antibiotic exposure to create germ-free lines (20 mg/L of tetracycline, streptomycin, and ampicillin) (Fig. 1). The concentration of antibiotics that removed the highest amount of gut bacteria without killing the host was determined experimentally (Additional file 1; Fig. A3). The genotypes were acclimated and synchronized for two generations in common garden conditions (16:8 light: dark regime, 20 ± 1 °C and fed 0.8 mg carbon/L of *C. vulgaris* daily) before they were exposed to glyphosate and Roundup. This practice is adopted to reduce interference from maternal and grandmaternal effects. After two generations in these conditions, clonal replicates aged 24–48 h from the second or following broods were randomly assigned to the experimental exposures in which host phenotype, transcriptome, and microbiome were assayed. Experimental animals were exposed to 1 mg/L of glyphosate and Roundup, corresponding to the drinking water Maximum Contaminant Level (MCL) set by the US Environmental Protection Agency [30]. Experimental conditions were as follows: 20 °C; 16:8 light:dark regime; the biological replicates of the 4 genotypes were kept in individual jars, fed daily with 0.8 mg carbon/L of *C. vulgaris*; the medium was renewed every second day; glyphosate and Roundup were refreshed at every medium change to maintain the xenobiont concentration constant throughout the experiment. Fitness-linked life history traits were measured in the time spanning an individual life cycle (until release of the second brood); transcriptome data were collected in the last instar to prevent developmental genes to overshadow the transcriptional response to the xenobionts.

### Fitness burden of Roundup and glyphosate

Size at maturity (distance between the head and the base of the tail spine), age at maturity (first time eggs were observed in the brood chamber), fecundity (total number of offspring released summing first and second brood), failed development of juveniles from first and second brood, and mortality were measured in the time spanning an individual life cycle (until release of the second brood) in control conditions (borehole water) and after chronic exposure to glyphosate (1 mg/L) and Roundup (Bayer) (1 mg/L). For size at maturity, all animals were measured after releasing their first brood into the brood pouch using ImageJ software (https://imagej.nih.gov/ij/index.html). Failed development was quantified as the percentage of dead or aborted embryos per genotype in the time spanning an individual life cycle as follows: (AE+DO)/(AE+DO+LO), where AE is aborted eggs, DO is dead offspring, and LO is live offspring (LO) [72]. Mortality rates per genotype were calculated with a survival model via the psm function in the rms R package V.3.3 [73]. A separate model was fitted to each treatment, in which the day of mortality and the mortality event were combined as the dependent variables (e.g., censoring). All mortality curves were plotted using the survplot function in the rms package in R v.3.3.3 [73].

The genotoxic effect of glyphosate and Roundup was measured using the comet assay [31]. The assay uses a microgel electrophoresis technique, in which a small number of cells are suspended in a thin agarose gel on a microscope slide, lysed, electrophoresed, and stained with a fluorescent DNA-binding dye. Cells with increased DNA damage display increased migration of chromosomal DNA from the nucleus toward the anode, which resembles the shape of a comet. We applied the comet assay to the hemolymph extracted from pools of ten non-exposed juveniles, used as reference, and on juvenile pools exposed for 72 h (corresponding to the last instar) to either glyphosate or Roundup, following standard protocols [31]. DNA damage was quantified on a minimum of 50 cells as the percentage of tail intensity (TI%). TI is the ratio between the total intensity of the tail and the total intensity of the comet (head and tail together) and is directly proportional to DNA damage [31].

We quantified the effects of genotype, treatment, and their interaction on the suite of life history traits using a univariate analysis of variance per trait (ANOVA). A two-way ANOVA was used to quantify the following terms: (1) genotype; significant differences among genotypes are indicative of genetic response; (2) treatment; a significant difference between control and treatment measures plastic response to a treatment; and (3) interaction term genotype by treatment. Before the analysis, the continuous dependent variables (fecundity, size and age of maturity, genotoxicity) were log transformed to meet the requirements of data normalization. In these analyses, linear mixed-effects models (LMMs) were used including clonal replicates as a random effect in R [74]. Because the standard deviation associated with the random effect was not significant in any of the models, we report results obtained using linear models with the *aov* function in R [75].

We visualized the main effect of treatment (glyphosate and Roundup) on fitness-linked life history traits (size at maturity, age at maturity, fecundity, and failed development) through univariate reaction norms, which describe the phenotypic expression of each genotype across treatments. We visualized the genotoxic effect of glyphosate and Roundup as the proportion of tail intensity per genotype.

### Host transcriptome response to Roundup and glyphosate

Total RNA was extracted from 20 last instar clones of each genotype across experimental conditions. Homogeneity of the transcriptional data is achieved by collecting whole animal tissue from pools of individuals at the same developmental stage. We used animals in the last instar, before they reached sexual maturity. The synchrony among the individuals, biological replicates, and genotypes is confirmed by a visual inspection of the *Daphnia* transparent body under a stereomicroscope, which allows to check for the presence of ovaries indicative of incipient sexual maturity.

The RNA advance kit (Beckman Coulter) was applied to flash-frozen tissue following the manufacturer’s instructions. Extracted RNA was quantified using a Nanodrop-8000 Spectrophotometer (Thermo Fisher ND-8000-GL) and integrity assessed on the Agilent Tapestation 2200 (Agilent G2964AA) with High Sensitivity RNA Screen Tapes (Agilent 5067- 5579). Total RNA (1 μg) was poly(A) selected using the NEBNext® Poly(A) mRNA Magnetic Isolation Module (New England Biolabs E7490L) and then converted in mRNA libraries using a NEBNext Ultra Directional RNA Library Prep Kit (New England Biolabs E7420L) and NEBnext Multiplex Oligos for Illumina Dual Index Primers (New England Biolabs E7600S), following the manufacturer guidelines. Sample handling was performed with the Biomek FxP work station (Beckman Coulter A31842). Constructed libraries were assessed for quality using the Tapestation 2200 (Agilent G2964AA) with High Sensitivity D1000 DNA Screen Tape (Agilent 5067-5584). Multiplexed libraries (100-bp paired end) were sequenced on a HiSeq4000 by EnviSion, BioSequencing, and BioComputing (University of Birmingham Enterprise) to obtain 5 M reads per samples and biological replicate. Sequenced reads quality was assessed using fastqc (v0.11.5) followed by multiqc (v1.5) (http://www.bioinformatics.babraham.ac.uk/projects/fastqc/). Transcripts were mapped onto the *D. magna* reference transcriptome [32, 76] and residual contaminant sequences, which may consist of residual gut bacteria and algae used as feedstock, were removed following blast searches in the NCBI database. The reads were then trimmed using Trimmomatic 0.36 [77] with the following parameters: (1) Illumina adapter cutoff with two seed mismatches, (2) palindrome clip threshold of 30 and a simple clip threshold of 10, (3) Phred quality score > 30; (4) minimum trimmed reads length of 50 bp. A read count matrix was created using Salmon [78]. Differential expression at gene and transcript level were quantified with DESeq2 1.20.0 in R [79]. Significantly differentially expressed genes, e.g., | logFC | > 2 were searched.

To gain insights into the regulatory patterns of uncharacterized genes, we performed a weighted gene co-expression network analysis with MODA 1.2.0 using the normalized expression data for all genes as described in [32]. We used MODA to identify gene clusters associated with either glyphosate or Roundup. The R package to run MODA can be found at https://www.bioconductor.org/packages/release/bioc/html/MODA.html; the R scripts used for our analyses are available on figshare: 10.6084/m9.figshare.13108049.v1. For each module, we performed a functional analysis of genes and pathways. We used gffread from GFF utilities to translate nucleotides into amino acid sequences. We then used InterProscan [80] for the functional analysis of protein sequences by classifying them into families and predicting their function based on domain information. To classify proteins, InterProscan uses predictive models provided by several databases including Pfam, PANTHER, CDD, GO, and KEGG. Further, we identified the pathways in which the proteins were enriched using two complementary approaches: (1) Panther [81] for the functional classification of genes from organisms across the Tree of Life, based on more than 900 genomes. For this analysis, *Daphnia pulex* ortholog genes were used as background. Significant enriched pathways and gene ontologies (GO) were selected after Bonferroni correction; (2) in-house scripts for the identification of KEGG pathways. We used OrthoDB to identify *D. pulex* orthologs from *D. magna* genes. We identified non-unique mappings for each *D. magna* gene on the KEGG pathways of *D. pulex*. We then used these data to weight the confusion matrix for Fisher’s exact test and chi-square test and corrected *P* values for enrichment analysis. Significant pathways were identified with FDR correction (Benjamini-Hochberg method, *P* < 0.05).

### Gut microbiota changes in response to glyphosate and Roundup

Our first task was to create a reference gut metagenome. Following this task, we determined the size and composition of the gut microbiota from gut colonization (48 h after birth) to last instar (144 h after birth). We then established whether the gut microbiota was genetically determined. The reference gut microbiota, the gut colonization dynamics and the origin of the gut microbiome are described in Additional file 1. These data informed the core experiment of this study, in which we analyzed the impact of glyphosate and Roundup on the established gut communities.

After the dynamics and genetic origin of the gut microbiome were established, we performed the core experiment of this study, in which the impact of glyphosate and Roundup on established gut communities was quantified on the same four genotypes used to measure fitness-linked life history traits and transcriptional profiles. In a split design, replicated clones were either treated with antibiotics and exposed to the treatments (germ-free) or directly exposed to the treatments (wild-type) (Fig. 1). At the last instar, up to 20 individuals per biological replicate per genotype and treatment were dissected to separate the guts from the animals. Dissected guts were flash frozen in liquid nitrogen and stored at – 80 °C.

Bacterial DNA for all experiments was extracted from flash frozen guts, using the PowerSoil DNA Isolation Kit MoBio (Thermo Fisher Scientific). Paired end 250 bp amplicon libraries for the V1 region of the 16S rRNA gene [82] were obtained using a 2-step PCR protocol with 96 × 96 dual tag barcoding to facilitate multiplexing and to reduce cross-talk between samples in downstream analyses [83]. PCR1 and PCR2 were conducted using Q5 HS High-Fidelity Master Mix (New England Biolabs) with the following cycling: (1) initial denaturation for 10 s at 98 °C, followed by 25 PCR cycles consisting of 30 s at 98 °C and 30 s at 64 °C or 66 °C followed by an extension step of 30 s at 72 °C. QCs were performed at both PCR steps. Excess primer dimers and dinucleotides from PCR1 were removed using Thermostable alkaline phosphatase (Promega) and Exonuclease I (New England Biolabs). PCR2 amplicons were purified using High Prep PCR magnetic beads (Auto Q Biosciences) and quantitated using a 200 PRO plate reader (TECAN) using qubit dsDNA HS solution (Invitrogen). A standard curve was created by running standards of known concentration on each plate against which sample concentration was determined. PCR2 amplicons were mixed in equimolar quantities (at a final concentration of 12 pmol) using a biomek FXp liquid handling robot (Beckman Coulter). Pool’s molarity was confirmed using a HS D1000 tapestation Screen Tape (Agilent) prior to PE 250 bp sequencing on an Illumina MiSeq platform to obtain 100,000 reads per sample and biological replica. The Illumina reads were filtered with cutadapt [84]. The primer sequences and the adapters were filtered and reads shorter than < 50 bp were removed. Read pairs with a minimum overlap of 50 bp were merged using PEAR v. 0.9.10 [85] and trimmed with FASTX-Toolkit v. 0.0.13 [86] to remove low-quality bases at both ends. The filtered and trimmed reads were analyzed with QIIME2 [87] to identify unique sub-operational taxonomic units (sOTUs), using the deblur/denoise-16S option and the reference sequences from the SILVA database (SSU release 132, with 99% sOTUs of full-length sequences [88]).

We measured differential sOTU abundances using DESeq2 v 1.22.1 in R [79]. We estimated the difference among microbiota communities with PERMANOVA using the package Vegan v 2.5-4 in R [34]. The diversity estimates for the microbiome were calculated using “Tools for microbiome analysis” in R v1.5.23 [89]. The sOTU composition in different experimental conditions was visualized using “Tools for microbiome analysis” [89] and phyloseq v1.26.0 [90]. Low coverage (< 500 reads) and low abundance samples (< 10 reads), as well as rare sOTUs (observed in < 2 samples), were excluded from the downstream analysis. Moreover, biological replicates showing less than 0.4 Pearson’s correlation within genotype were excluded from the analyses (ca. 111 sOTUs per sample; data not shown).

To characterize the core gut microbial community in *D. magna*, where “core” is defined as a taxonomic unit with abundance larger than 1% within treatment, we used the taxonomic classifier SILVA 132 with 99% sOTUs similarity [91]. Taxonomic assignment for each taxon was completed in QIIME2 [92] at different taxonomic levels, from genus to phylum. The differences between mean values of relative abundances of each taxa at various taxonomic levels were tested by two-way ANOVA and Tukey HSD in R [75]. We calculated Alpha (Shannon, Evenness, observed sOTUs number, and Phylogenetic Distance) and Beta (Jaccard, Bray-Curtis, unweighted UniFrac, and weighted UniFrac) diversity between treatments and genotypes after correcting for uneven depths of sequencing using QIIME2 [92]. All samples were randomly resampled to achieve normalization to the samples with the smallest number of reads (13,439) prior to calculating diversity indices. Significant differences among genotypes and treatments in both indices were calculated using the Kruskal-Wallis and the PERMANOVA test. Further, differences between treatments and genotypes (13.2/13.5-1/3.5-15/P-IT) were quantified using Adonis, Anosim, and MRPP using the Vegan package in R [93]. We predicted the function of gut microbial communities in *D. magna* in presence and absence of treatments (glyphosate and Roundup) with the Tax4Fun package in R [94], using the randomly resampled reads used for the estimates of Alpha and Beta diversity. We assessed function similarity between treatments using the Jaccard and Bray-Curtis indexes. Statistically significant differences in functional composition were quantified with Adonis, Anosim, and MRPP. Furthermore, we applied two-way ANOVA and Turkey test to identify significant KEGG Orthology (KO) between pairs of genotypes within treatment and between treatments and control. Significantly different KO terms were mapped to KEGG pathways and enriched pathways were identified by Fisher’s exact test.

### Correlations between transcriptome and microbiome changes

We applied machine learning-based statistical analysis to identify correlations between genome-wide transcriptional changes and microbiota composition induced by glyphosate and Roundup chronic exposures. The workflow and scripts for these analyses can be found at: 10.6084/m9.figshare.13108049.v1. Before the analysis, invariant genes and genes with high proportion of missing counts across treatments were removed. To reduce data sparsity, sOTUs were retained at the genus level when they were present in at least 60% of samples across treatments.

We applied Random Forest models (ensemble of decision trees), the Random Forest classifier, and the Random Forest regression [35]. These models provide reliable predictions even when handling noisy and sparse data [95]. Furthermore, they capture information linked to the interactions among different dataset features [96].

In the Random Forest Regression models, sOTUs were the dependent variables and genes were the independent variables. We regressed the two variables separately for Roundup and glyphosate in a highly replicated manner (each model was run 80 times). Each tree generated by the ensemble model was fitted to minimize mean-squared error (MSE), using cross-validation hyperparameter tuning method (https://scikit-learn.org/stable/modules/grid_search.html) with 100 random combinations of parameters. The features with the lowest average MSE across trees were selected if they contributed at least 20% to the total model features. This threshold ensures that at least one gene of major effect contributes to the model. The gene modules identified in this analysis were used in a functional analysis to identify the pathways in which the proteins were enriched using two complementary approaches as above: (1) Panther [81] for the functional classification of genes from organisms across the Tree of Life, based on more than 900 genomes. For this analysis, *D. pulex* orthologous genes were used as background. Significant enriched pathways and gene ontologies (GO) were selected after Bonferroni correction; (2) in-house scripts for the identification of KEGG pathways. We used OrthoDB to identify *D. pulex* orthologs from *D. magna* genes. We identified non-unique mappings for each *D. magna* gene on the KEGG pathways of *D. pulex*. We then use these data to weight the confusion matrix for Fisher’s exact test and chi-square test and correct *P* values for enrichment analysis. Significant pathways were identified with FDR correction (Benjamini-Hochberg method, *P* < 0.05).

## Supplementary Information

**Additional file 1.**

## Data Availability

Fitness and genotoxic data associated with this study are deposited in the DRYAD databank at the following entry: doi:10.5061/dryad.mcvdncjws. Transcriptome data supporting the findings of this study are published under the International Nucleotide Sequence Database Collaboration BioProject PRJNA606209. Microbiome sequence data supporting the findings of this study are available at the NCBI entry PRJNA606209. All codes and software used in this paper are publicly available. Scripts and workflows are available at: 10.6084/m9.figshare.13108049.v1.

## References

[1] Blair A (2015). Carcinogenicity of tetrachlorvinphos, parathion, malathion, diazinon, and glyphosate. Lancer Oncology.

[2] Wang L (2019). Glyphosate induces benign monoclonal gammopathy and promotes multiple myeloma progression in mice. J Hematol Oncol.

[3] Samsel A, Seneff S (2013). Glyphosate, pathways to modern diseases II: celiac sprue and gluten intolerance. Interdiscip Toxicol.

[4] Pu Y (2020). Maternal glyphosate exposure causes autism-like behaviors in offspring through increased expression of soluble epoxide hydrolase. Proc Natl Acad Sci U S A.

[5] Trevan JW (1927). The error of determination of toxicity. Proc R Soc.

[6] ECHA (2016). Guidance on information requirements and chemical safety assessment.

[7] Bopp SK (2019). Regulatory assessment and risk management of chemical mixtures: challenges and ways forward. Crit Rev Toxicol.

[8] Pollegioni L, Schonbrunn E, Siehl D (2011). Molecular basis of glyphosate resistance - different approaches through protein engineering. Febs J.

[9] Lynch JB, Hsiao EY (2019). Microbiomes as sources of emergent host phenotypes. Science.

[10] Motta EVS, Raymann K, Moran NA (2018). Glyphosate perturbs the gut microbiota of honey bees. Proc Natl Acad Sci U S A.

[11] Shehata AA, Schrodl W, Aldin AA, Hafez HM, Kruger M (2013). The effect of glyphosate on potential pathogens and beneficial members of poultry microbiota in vitro. Curr Microbiol.

[12] Krüger M, Schrödl W, Neuhaus J, Shehata A (2013). Journal of Environmental & Analytical Toxicology. J Environ Anal Toxicol.

[13] Carman JA (2013). A long-term toxicology study on pigs fed a combined genetically modified (GM) soy and GM maize diet. J Org Syst.

[14] Yang X (2019). Effects of the glyphosate-based herbicide roundup on the survival, immune response, digestive activities and gut microbiota of the Chinese mitten crab, Eriocheir sinensis. Aquat Toxicol.

[15] Dechartres J (2019). Glyphosate and glyphosate-based herbicide exposure during the peripartum period affects maternal brain plasticity, maternal behaviour and microbiome. J Neuroendocrinol.

[16] Sihtmae M (2013). Ecotoxicological effects of different glyphosate formulations. Appl Soil Ecol.

[17] Van Bruggen AHC (2018). Environmental and health effects of the herbicide glyphosate. Sci Total Environ.

[18] Grandcoin A, Piel S, Baures E (2017). AminoMethylPhosphonic acid (AMPA) in natural waters: its sources, behavior and environmental fate. Water Res.

[19] Birch H, Mikkelsen PS, Jensen JK, Lutzhoft HCH (2011). Micropollutants in stormwater runoff and combined sewer overflow in the Copenhagen area, Denmark. Water Sci Technol.

[20] Noori JS, Dimaki M, Mortensen J, Svendsen WE. Detection of glyphosate in drinking water: a fast and direct detection method without sample pretreatment. Sensors Basel. 2018;18, ARTN 2961. 10.3390/s18092961. 10.3390/s18092961 PMC6163928 30189680

[21] Székács A, Darvas B. Re-registration challenges of glyphosate in the European Union. Front Environ Sci. 2018. 10.3389/fenvs.2018.00078.

[22] Colbourne JK (2011). The ecoresponsive genome of Daphnia pulex. Science.

[23] Altshuler I (2011). An integrated multi-disciplinary approach for studying multiple stressors in freshwater ecosystems: Daphnia as a model organism. Integr Comp Biol.

[24] Miner BE, De Meester L, Pfrender ME, Lampert W, Hairston NG (2012). Linking genes to communities and ecosystems: Daphnia as an ecogenomic model. P Roy Soc B-Biol Sci.

[25] Ebert D. Ecology, epidemiology, and evolution of parasitism in Daphnia. Bethesda: National Library of Medicine (US), National Center for Biotechnology; 2005.

[26] Cambronero Cuenca M, Orsini L (2018). Resurrection of dormant Daphnia magna: protocol and applications. JoVE.

[27] Kerfoot WC, Weider LJ (2004). Experimental paleoecology (resurrection ecology): Chasing Van Valen’s Red Queen hypothesis. Limnol Oceanogr.

[28] Cambronero CM, Beasley J, Kissane S, Orsini L (2018). Evolution of thermal tolerance in multifarious environments. Mol Ecol.

[29] Cambronero CM (2018). Predictability of the impact of multiple stressors on the keystone species Daphnia. Sci Rep-Uk.

[30] Agency USEP (1995). National primary drinking water regulation: Glyphosate.

[31] Speit G, Hartmann A. DNA Repair Protocols: Mammalian Systems. Totowa: Humana Press Inc; 2006.

[32] Orsini L (2018). Early transcriptional response pathways in Daphnia magna are coordinated in networks of crustacean-specific genes. Mol Ecol.

[33] Fabregat A (2018). The Reactome Pathway Knowledgebase. Nucleic Acids Res.

[34] Oksanen J (2011). Multivariate analysis of ecological communities in R: vegan tutorial.

[35] Breiman L (2001). Random Forests. Mach Learn.

[36] Jacquemont C, Taniguchi T (2007). The Fanconi anemia pathway and ubiquitin. BMC Biochem.

[37] Smith CM, Vera MKM, Bhandari RK (2019). Developmental and epigenetic effects of Roundup and glyphosate exposure on Japanese medaka (Oryzias latipes). Aquat Toxicol.

[38] Webster TMU, Laing LV, Florance H, Santos EM (2014). Effects of glyphosate and its formulation, roundup, on reproduction in zebrafish (Danio rerio). Environ Sci Technol.

[39] Alvarez-Moya C (2014). Comparison of the in vivo and in vitro genotoxicity of glyphosate isopropylamine salt in three different organisms. Genet Mol Biol.

[40] Clements C, Ralph S, Petras M (1997). Genotoxicity of select herbicides in Rana catesbeiana tadpoles using the alkaline single-cell gel DNA electrophoresis (comet) assay. Environ Mol Mutagen.

[41] Poletta GL, Larriera A, Kleinsorge E, Mudry MD (2009). Genotoxicity of the herbicide formulation Roundup (R) (glyphosate) in broad-snouted caiman (Caiman latirostris) evidenced by the Comet assay and the Micronucleus test. Mutat Res Gen Tox En.

[42] Nwani CD, Nagpure NS, Kumar R, Kushwaha B, Lakra WS (2013). DNA damage and oxidative stress modulatory effects of glyphosate-based herbicide in freshwater fish, Channa punctatus. Environ Toxicol Phar.

[43] Moreno NC, Sofia SH, Martinez CBR (2014). Genotoxic effects of the herbicide Roundup Transorb (R) and its active ingredient glyphosate on the fish Prochilodus lineatus. Environ Toxicol Phar.

[44] Schaumburg LG, Siroski PA, Poletta GL, Mudry MD (2016). Genotoxicity induced by Roundup (R) (Glyphosate) in tegu lizard (Salvator merianae) embryos. Pestic Biochem Phys.

[45] Hong YH, Yang XZ, Huang Y, Yan GW, Cheng YX (2018). Assessment of the oxidative and genotoxic effects of the glyphosate-based herbicide roundup on the freshwater shrimp, Macrobrachium nipponensis. Chemosphere.

[46] Gill JPK, Sethi N, Mohan A, Datta S, Girdhar M (2018). Glyphosate toxicity for animals. Environ Chem Lett.

[47] Kier LD, Kirkland DJ (2013). Review of genotoxicity studies of glyphosate and glyphosate-based formulations. Crit Rev Toxicol.

[48] Asselman J (2015). Conserved transcriptional responses to cyanobacterial stressors are mediated by alternate regulation of paralogous genes in Daphnia. Mol Ecol.

[49] Pereira JL, et al. Gene transcription in Daphnia magna: effects of acute exposure to a carbamate insecticide and an acetanilide herbicide. Aquat Toxicol. 2010; in press. 10.1016/j.aquatox.2009.12.023 20092900

[50] Tsui MTK, Wang WX (2005). Multigenerational acclimation of Daphnia magna to mercury: relationships between biokinetics and toxicity. Environ Toxicol Chem.

[51] Tsui MT-K, Wang WX (2009). Biokinetics and tolerance development of toxic metals in Daphnia magna. Environ Toxicol Chem.

[52] Webster TMU, Santos EM. Global transcriptomic profiling demonstrates induction of oxidative stress and of compensatory cellular stress responses in brown trout exposed to glyphosate and Roundup. Bmc Genomics. 2015;16, doi: ARTN 32. 10.1186/s12864-015-1254-5. 10.1186/s12864-015-1254-5 PMC4318436 25636363

[53] Mesnage, R. *et al.* Transcriptome profile analysis reflects rat liver and kidney damage following chronic ultra-low dose Roundup exposure. Environ Health Glob (14, pg 70, 2015). 10.1186/s12940-017-0236-2. 16. ARTN 28 (2017). 10.1186/s12940-015-0056-1 PMC4549093 26302742

[54] Simoes T, et al. An integrative omics approach to unravel toxicity mechanisms of environmental chemicals: effects of a formulated herbicide. Sci Rep-Uk. 2018:8. 10.1038/s41598-018-29662-6 ARTN 11376. 10.1038/s41598-018-29662-6 PMC6063884 30054531

[55] Snijders AM (2016). Influence of early life exposure, host genetics and diet on the mouse gut microbiome and metabolome. Nat Microbiol.

[56] Kokou F (2019). Core gut microbial communities are maintained by beneficial interactions and strain variability in fish. Nat Microbiol.

[57] Benjamino J, Lincoln S, Srivastava R, Graf J (2018). Low-abundant bacteria drive compositional changes in the gut microbiota after dietary alteration. Microbiome.

[58] Zhan H, Feng Y, Fan X, Chen S (2018). Recent advances in glyphosate biodegradation. Appl Microbiol Biotechnol.

[59] Balthazor TM, Hallas LE (1986). Glyphosate-degrading microorganisms from industrial activated sludge. Appl Environ Microbiol.

[60] Liu CM, McLean PA, Sookdeo CC, Cannon FC (1991). Degradation of the herbicide glyphosate by members of the family Rhizobiaceae. Appl Environ Microbiol.

[61] Menendez E, Carro L, Giri B, Prasad R, Wu QS, Varma A (2019). Biofertilizers for Sustainable Agriculture and Environment vol. Soil Biology.

[62] Binda C (2018). Actinobacteria: a relevant minority for the maintenance of gut homeostasis. Dig Liver Dis.

[63] Rinninella E (2019). What is the healthy gut microbiota composition? A changing ecosystem across age, environment, diet, and diseases. Microorganisms.

[64] Azad MAK, Sarker M, Li T, Yin J (2018). Probiotic species in the modulation of gut microbiota: an overview. Biomed Res Int.

[65] Cheng SY (2005). Thyroid hormone receptor mutations and disease: beyond thyroid hormone resistance. Trends Endocrinol Metab.

[66] Clement K (1998). A mutation in the human leptin receptor gene causes obesity and pituitary dysfunction. Nature.

[67] Komiya Y, Habas R (2008). Wnt signal transduction pathways. Organogenesis.

[68] Jacob L, Lum L (2007). Hedgehog signaling pathway in Drosophila. Sci STKE.

[69] Jia Y, Wang Y, Xie J (2015). The Hedgehog pathway: role in cell differentiation, polarity and proliferation. Arch Toxicol.

[70] Liu Y (2018). Critical role of FOXO3a in carcinogenesis. Mol Cancer.

[71] Massague J (2012). TGFbeta signalling in context. Nat Rev Mol Cell Biol.

[72] Pellegri V. Ecological risk assessment and development of innovative strategies for monitoring the quality of water bodies: application of a new integrated approach in a pilot basin: PhD in Biology thesis. University of Padova; 2015.

[73] Team, R Core. 2019. R: A language and environment for statistical computing. In R Foundation for Statistical Computing. Vienna, Austria.

[74] Pinheiro, J., Bates, D., DebRoy, S. & Sarkar, D. 1-137 (https://CRAN.R-project.org/package=nlme, 2018).

[75] Team, R. C. (https://www.r-project.org/. Vienna. 2018).

[76] Orsini L (2016). Daphnia magna transcriptome by RNA-Seq across 12 environmental stressors. Sci Data.

[77] Bolger AM, Lohse M, Usadel B (2014). Trimmomatic: a flexible trimmer for Illumina sequence data. Bioinformatics.

[78] Patro R, Duggal G, Love MI, Irizarry RA, Kingsford C (2017). Salmon provides fast and bias-aware quantification of transcript expression. Nat Methods.

[79] Love MI, Huber W, Anders S (2014). Moderated estimation of fold change and dispersion for RNA-seq data with DESeq2. Genome Biol.

[80] Jones P (2014). InterProScan 5: genome-scale protein function classification. Bioinformatics.

[81] Mi H (2019). Protocol update for large-scale genome and gene function analysis with the PANTHER classification system (v.14.0). Nat Protoc.

[82] Chakravorty S, Helb D, Burday M, Connell N, Alland D (2007). A detailed analysis of 16S ribosomal RNA gene segments for the diagnosis of pathogenic bacteria. J Microbiol Methods.

[83] MacConaill LE (2018). Unique, dual-indexed sequencing adapters with UMIs effectively eliminate index crosstalk and significantly improve sensitivity of massively parallel sequencing. BMC Genomics.

[84] Martin M (2011). Cutadapt removes adapter sequences from high-throughput sequencing reads. EMBnet J.

[85] Zhang J, Kobert K, Flouri T, Stamatakis A (2014). PEAR: a fast and accurate Illumina Paired-End reAd mergeR. Bioinformatics.

[86] FASTX-Toolkit (http://hannonlab.cshl.edu/fastx_toolkit. 2010).

[87] Bolyen E (2018). QIIME 2: Reproducible, interactive, scalable, and extensible microbiome data science. PeerJ Preprints.

[88] Pruesse E (2007). SILVA: a comprehensive online resource for quality checked and aligned ribosomal RNA sequence data compatible with ARB. Nucleic Acids Res.

[89] Lahti, L., Shetty, S. & et al. (https://microbiome.github.io/microbiome/. 2017).

[90] McMurdie PJ, Holmes S (2013). phyloseq: an R package for reproducible interactive analysis and graphics of microbiome census data. PLoS One.

[91] Yilmaz P (2014). The SILVA and “All-species Living Tree Project (LTP)” taxonomic frameworks. Nucleic Acids Res.

[92] Bolyen E (2019). Reproducible, interactive, scalable and extensible microbiome data science using QIIME 2. Nat Biotechnol.

[93] Dixon P (2003). VEGAN, a package of R functions for community ecology. J Veg Sci.

[94] Asshauer KP, Wemheuer B, Daniel R, Meinicke P (2015). Tax4Fun: predicting functional profiles from metagenomic 16S rRNA data. Bioinformatics.

[95] Diaz-Uriarte R, de Andres SA. Gene selection and classification of microarray data using random forest. Bmc Bioinform. 2006;7. 10.1186/1471-2105-7-3 Artn 3. 10.1186/1471-2105-7-3 PMC1363357 16398926

[96] Chen X, Ishwaran H (2012). Random forests for genomic data analysis. Genomics.

