# Supplementary material for: Roundup causes embryonic development failure and alters metabolic pathways and gut microbiota functionality in non-target species
Source: Microbiome. 2020 Dec 15;8:170. doi: 10.1186/s40168-020-00943-5 (PMC7780628; doi:10.1186/s40168-020-00943-5)

**Figure S1.** **Univariate reaction norms**. Univariate responses to Glyphosate and Roundup in four genotypes of *D. magna*. Genotype reaction norms based on biological replicates means (n = 3) and SD, are shown for size and age at maturity, fecundity, calculated as the total number of juveniles from the first two broods, and failed development, quantified as the percentage of dead or aborted embryos per genotype in the time spanning an individual life cycle. The reaction norms support statistical analyses in Table 1.


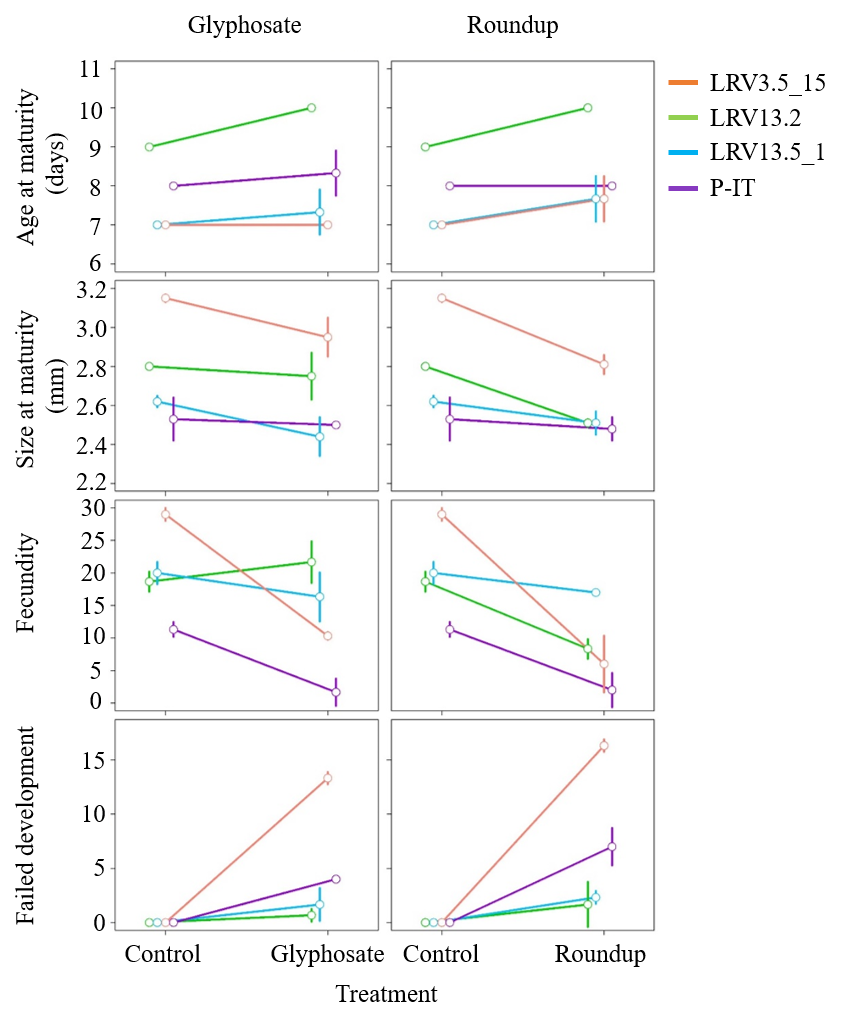

Supplement: Supplementary file 2 — Additional file 1. [file 40168_2020_943_MOESM1_ESM.zip › Suppa et al_ Fig.S1_ESM.docx]
