# Supplementary material for: Roundup causes embryonic development failure and alters metabolic pathways and gut microbiota functionality in non-target species
Source: Microbiome. 2020 Dec 15;8:170. doi: 10.1186/s40168-020-00943-5 (PMC7780628; doi:10.1186/s40168-020-00943-5)
Supplement: Supplementary file 2 — Additional file 1. [file 40168_2020_943_MOESM1_ESM.zip › Suppa et al_Fig.S2_ESM.docx]

**Figure S2. Mortality.** Mortality per genotype across biological replicates and exposures, Glyphosate, Roundup and control, measured over 21 days. Data are shown for wild type animals not treated with antibiotics. Mortality was calculated with survival analysis in R using the rms package (<https://cran.r-project.org/web/packages/rms/rms.pdf>). Genotypes are color coded as in Figure S1.

**
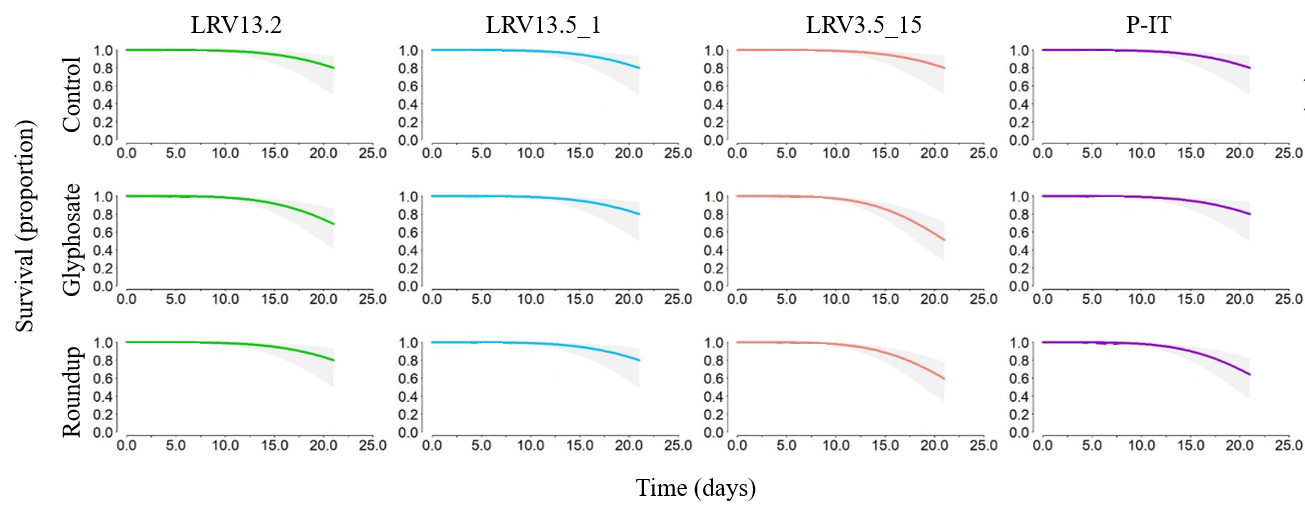
**
