# Supplementary material for: Roundup causes embryonic development failure and alters metabolic pathways and gut microbiota functionality in non-target species
Source: Microbiome. 2020 Dec 15;8:170. doi: 10.1186/s40168-020-00943-5 (PMC7780628; doi:10.1186/s40168-020-00943-5)

**Figure S3. DNA damage**

Quantification of DNA damage via the Comet assay are shown per genotype with variance across the biological replicates. The intensity is directly proportional to DNA damage. The supporting statistics to these plots are in Table 1.


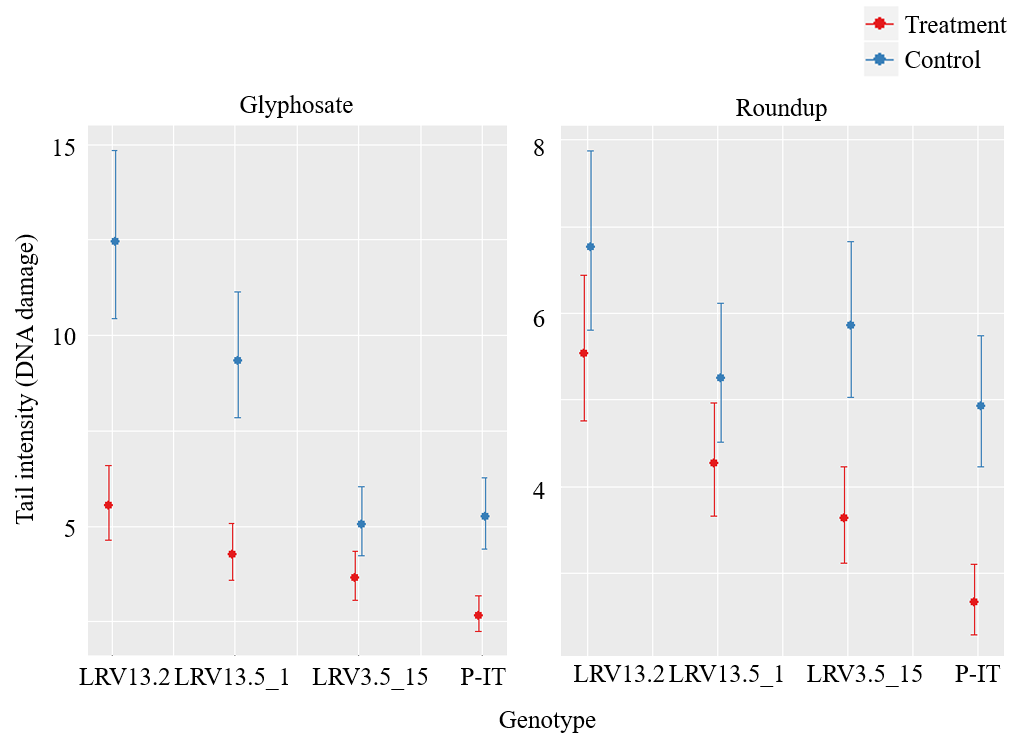

Supplement: Supplementary file 2 — Additional file 1. [file 40168_2020_943_MOESM1_ESM.zip › Suppa et al_Fig.S3_ESM.docx]
