# Supplementary material for: Roundup causes embryonic development failure and alters metabolic pathways and gut microbiota functionality in non-target species
Source: Microbiome. 2020 Dec 15;8:170. doi: 10.1186/s40168-020-00943-5 (PMC7780628; doi:10.1186/s40168-020-00943-5)
Supplement: Supplementary file 2 — Additional file 1. [file 40168_2020_943_MOESM1_ESM.zip › Suppa etal_Fig.S4_ESM.docx]

**Figure S4. PCA**

PCA plots including whole transcriptome data on the four genotypes and their replicates used in this study across all treatments. The genotype colour code is as in Figure S1.

**
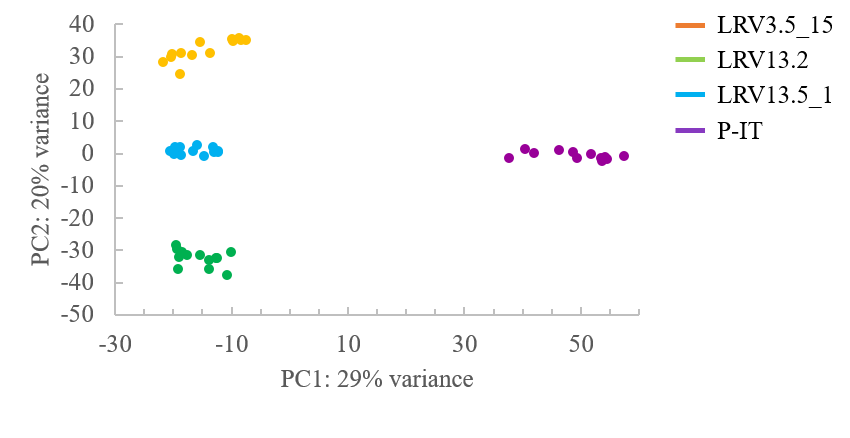
**
