# Supplementary material for: Roundup causes embryonic development failure and alters metabolic pathways and gut microbiota functionality in non-target species
Source: Microbiome. 2020 Dec 15;8:170. doi: 10.1186/s40168-020-00943-5 (PMC7780628; doi:10.1186/s40168-020-00943-5)

**Figure S5. MDS plot.** Hierarchical clustering of OTUs is shown for the clonal replicates of the four genotypes: LRV3.5_15, LRV13.5_1, LRV13.2 and P-IT. Open symbols indicate treatments: Roundup (triangle), Glyphosate (square) and control (circle). Close symbols indicate the treatments combined with antibiotic exposure. The genotypes color code is as in Figure S1. The OTU for the borehole microbial composition is also shown. BW – Borehole Water; Co – Control; Co+A – Control with antibiotic treatment; Gly – Glyphosate; Gly+A - Glyphosate with antibiotic treatment; Rou – Roundup; Rou+A – Roundup with antibiotic treatment.


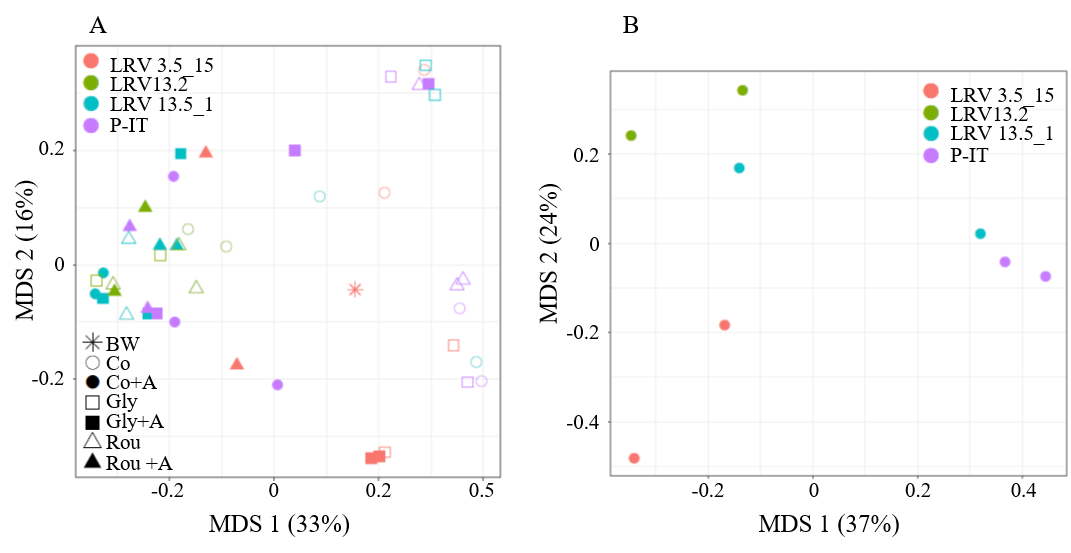

Supplement: Supplementary file 2 — Additional file 1. [file 40168_2020_943_MOESM1_ESM.zip › Suppa etal_Fig.S5_ESM.docx]
