# Supplementary material for: Roundup causes embryonic development failure and alters metabolic pathways and gut microbiota functionality in non-target species
Source: Microbiome. 2020 Dec 15;8:170. doi: 10.1186/s40168-020-00943-5 (PMC7780628; doi:10.1186/s40168-020-00943-5)
Supplement: Supplementary file 2 — Additional file 1. [file 40168_2020_943_MOESM1_ESM.zip › Suppa etal_Table.S4_ESM.docx]

**Table S4. PERMANOVA on beta diversity.** Permutational Multivariate Analysis of Variance using Jaccard and Bray Curtis similarities indices to estimate beta diversity between treatments and control across genotypes and between pairs of genotypes. Averaged values across biological replicates within genotype are shown. Significant P-values at 0.05 are in bold.

| Beta diversity index | Jaccard distance | | | | Bray Curtis distance | | | |
| --- | --- | --- | --- | --- | --- | --- | --- | --- |
| Dataset | OTUs profile | | KOs profile | | OTUs profile | | KOs profile | |
| model: treatment + strainID +  treatment * strainID | F.Model | Pr(>F) | F.Model | Pr(>F) | F.Model | Pr(>F) | F.Model | Pr(>F) |
| Treatment | 0.935 | 0.638 | 1.281 | 0.302 | 0.935 | 0.638 | 1.281 | 0.302 |
| StrainID | 0.916 | 0.751 | 1.495 | 0.232 | 0.916 | 0.751 | 1.495 | 0.232 |
| Treatment * StrainID | 1.055 | 0.232 | 0.424 | 0.925 | 1.055 | 0.232 | 0.424 | 0.925 |
| model: treatment | 0.915 | 0.706 | 0.496 | 0.728 | 0.757 | 0.695 | 1.365 | 0.266 |
| Control vs. Glyphosate | 0.919 | 0.617 | 0.242 | 0.740 | 0.954 | 0.437 | 0.885 | 0.383 |
| Control vs. Roundup | 0.894 | 0.665 | 1.216 | 0.306 | 0.538 | 0.796 | 0.499 | 0.602 |
| Glyphosate vs. Roundup | 0.931 | 0.591 | 2.010 | 0.162 | 0.781 | 0.596 | 0.005 | 0.917 |
| model: strainID | 1.436 | **0.004** | 3.577 | **0.006** | 1.949 | **0.011** | 1.645 | 0.160 |
| LRV13.2 vs LRV13.5_1 | 0.909 | 0.704 | 1.156 | 0.527 | 0.860 | 0.486 | 0.770 | 0.439 |
| LRV13.2 vs LRV3.5_15 | 1.714 | **0.015** | 0.139 | 0.847 | 3.282 | **0.013** | 4.978 | **0.019** |
| LRV13.2 vs P-IT | 1.727 | **0.011** | 1.000 | 0.292 | 1.745 | 0.111 | 7.969 | **0.010** |
| LRV13.5_1 vs LRV3.5_15 | 1.612 | **0.022** | 2.339 | **0.050** | 2.077 | **0.043** | 2.527 | 0.094 |
| LRV13.5_1 vs P-IT | 1.658 | **0.008** | 10.717 | **0.004** | 2.040 | 0.057 | 4.010 | 0.054 |
| LRV3.5_15 vs P-IT | 0.996 | 0.415 | 0.155 | 0.820 | 1.726 | 0.124 | 1.224 | 0.300 |
