# Supplementary material for: Roundup causes embryonic development failure and alters metabolic pathways and gut microbiota functionality in non-target species
Source: Microbiome. 2020 Dec 15;8:170. doi: 10.1186/s40168-020-00943-5 (PMC7780628; doi:10.1186/s40168-020-00943-5)
Supplement: Supplementary file 2 — Additional file 1. [file 40168_2020_943_MOESM1_ESM.zip › Suppa etal_Table.S5_ESM.docx]

**Table S5. Taxa relative abundance across genotypes.** Relative abundance of bacterial taxa, quantified at Family and Genus level, across the four genotypes and biological replicates. Calculations were obtained using the genotype P-IT as reference. Core taxa, taxa with abundance larger than 1% across treatments and detected in at least 90% of the samples, are underlined. *P*-values are adjusted using the Benjamini-Hochberg method with a false discovery rate of 0.05. Significant values are in bold.

| Phylum | Family | LRV13.5_1 | LRV13.2 | LRV3.5_15 | P-IT | Genus | LRV13.5_1 | LRV13.2 | LRV3.5_15 | P-IT |
| --- | --- | --- | --- | --- | --- | --- | --- | --- | --- | --- |
| *Proteobacteria* | *Moraxellaceae* | 23.1 | **10.3*** | 21.1 | 48.4 | *Acinetobacter* | 23.1 | **10.0*** | 21.0 | 48.4 |
|  | *Pseudomonadaceae* | 14.0 | 11.2 | 23.5 | 11.5 | *Pseudomonas* | 14.0 | 11.2 | 23.5 | 11.5 |
|  | *Burkholderiaceae* | 2.7 | **7.1**** | 18.1 | 12.1 | *Acidovorax* | **0.5*** | 2.3 | 9.5 | 5.4 |
|  |  |  |  |  |  | *Limnohabitans* | **0.9**** | **2.9*** | 5.7 | 5.3 |
|  |  |  |  |  |  | *Ralstonia* | 0.0 | 0.0 | 1.6 | 0.1 |
|  | *Legionellaceae* | 1.6 | 0.5 | 0.1 | 0.1 | *Legionella* | **1.6*** | 0.5 | 0.1 | 0.1 |
|  | *Beijerinckiaceae* | 4.3 | **1.5**** | 0.7 | 0.7 |  |  |  |  |  |
|  | *Rhizobiaceae* | 0.7 | **0.8***** | 0.8 | 0.7 | *Methylobacterium* | 2.8 | 0.9 | 0.3 | 0.4 |
|  | *Xanthobacteraceae* | 0.3 | 1.3 | 0.3 | 0.3 |  |  |  |  |  |
|  | *Sphingomonadaceae* | 4.8 | 2.2 | 10.2 | 2.9 | *Sphingoaurantiacus* | 1.1 | 0.0 | 0.0 | 0.0 |
|  |  |  |  |  |  | *Sphingobium* | 1.0 | 1.3 | 9.7 | 1.8 |
|  |  |  |  |  |  | *Sphingomonas* | 2.4 | 0.9 | 0.2 | 0.7 |
|  | *Caulobacteraceae* | 1.0 | 0.5 | 0.9 | 1.2 |  |  |  |  |  |
| *Actinobacteria* | *Propionibacteriaceae* | 20.8 | 26.8 | 5.8 | 6.5 | *Cutibacterium* | 20.5 | 26.8 | 5.7 | 6.4 |
|  | *Microbacteriaceae* | 1.9 | 4.0 | 5.4 | 5.0 | *Galbitalea* | 1.4 | **3.8*** | 5.0 | 2.5 |
|  |  |  |  |  |  | *Microbacterium* | 0.1 | 0.1 | 0.3 | 1.7 |
|  | *Corynebacteriaceae* | 6.5 | 11.3 | 1.4 | 1.4 | *Corynebacterium 1* | 6.4 | 11.2 | 1.3 | 1.3 |
|  | *Nocardiaceae* | 3.7 | **4.1**** | 3.3 | 3.2 | *Rhodococcus* | 3.3 | **3.3**** | 3.0 | 2.8 |
| *Firmicutes* | *Streptococcaceae* | 1.0 | 0.8 | 0.3 | 0.3 | *Streptococcus* | 1.0 | 0.8 | 0.3 | 0.3 |
|  | *Staphylococcaceae* | 3.4 | 3.1 | 0.2 | 0.6 | *Staphylococcus* | 3.4 | 3.1 | 0.2 | 0.6 |
| *Bacteroidetes* | *Prevotellaceae* | 2.6 | 1.6 | 0.0 | 0.0 | *Alloprevotella* | 2.4 | 0.3 | 0.0 | 0.0 |
|  | *Flavobacteriaceae* | 0.0 | **5.2***** | 3.4 | 1.6 | *Flavobacterium* | **0.0**** | **5.2**** | 3.4 | 1.6 |
| sum |  | 92.2 | 92.2 | 95.5 | 96.4 |  | 85.7 | 84.6 | 90.9 | 90.9 |
