# Supplementary material for: Roundup causes embryonic development failure and alters metabolic pathways and gut microbiota functionality in non-target species
Source: Microbiome. 2020 Dec 15;8:170. doi: 10.1186/s40168-020-00943-5 (PMC7780628; doi:10.1186/s40168-020-00943-5)
Supplement: Supplementary file 2 — Additional file 1. [file 40168_2020_943_MOESM1_ESM.zip › Suppa etal_Table.S7_ESM.docx]

**Table S7**. KEGG enriched pathways were identified by Fisher Exact (P<0.05) test following exposure to Glyphosate and Roundup. Pathways differentially enriched between Glyphosate and Roundup are also shown. Significant changes are in bold.

| Pathway | Glyphosate vs Control | Roundup vs Control | Roundup vs Glyphosate | Description | Class | Subclass |
| --- | --- | --- | --- | --- | --- | --- |
| map00010 | 0.055 | 1.000 | **0.035** | Glycolysis / Gluconeogenesis | Metabolism | Carbohydrate metabolism |
| map00051 | **0.001** | 1.000 | 1.000 | Fructose and mannose metabolism | Metabolism | Carbohydrate metabolism |
| map00500 | **0.001** | 1.000 | 1.000 | Starch and sucrose metabolism | Metabolism | Carbohydrate metabolism |
| map00520 | **0.001** | 1.000 | **0.035** | Amino sugar and nucleotide sugar metabolism | Metabolism | Carbohydrate metabolism |
| map00620 | **0.001** | 1.000 | **0.035** | Pyruvate metabolism | Metabolism | Carbohydrate metabolism |
| map00640 | **0.001** | 1.000 | **0.035** | Propanoate metabolism | Metabolism | Carbohydrate metabolism |
| map00650 | 0.055 | 1.000 | **0.035** | Butanoate metabolism | Metabolism | Carbohydrate metabolism |
| map00720 | **0.001** | **0.006** | 1.000 | Carbon fixation pathways in prokaryotes | Metabolism | Energy metabolism |
| map02010 | 0.055 | 1.000 | **0.035** | ABC transporters | Environmental Information Processing | Membrane transport |
| map02060 | **0.000** | 1.000 | 1.000 | Phosphotransferase system (PTS) | Environmental Information Processing | Membrane transport |
| map02020 | 0.055 | **0.006** | **0.035** | Two-component system | Environmental Information Processing | Signal transduction |
| map00970 | 1.000 | 1.000 | **0.035** | Aminoacyl-tRNA biosynthesis | Genetic Information Processing | Translation |
| map00230 | 0.055 | 1.000 | **0.035** | Purine metabolism | Metabolism | Nucleotide metabolism |
| map00071 | 1.000 | 1.000 | **0.035** | Fatty acid degradation | Metabolism | Lipid metabolism |
| map00362 | 1.000 | 1.000 | **0.035** | Benzoate degradation | Metabolism | Xenobiotics biodegradation and metabolism |
| map00930 | 1.000 | 1.000 | **0.035** | Caprolactam degradation | Metabolism | Xenobiotics biodegradation and metabolism |
| map05203 | 0.055 | 1.000 | **0.035** | Viral carcinogenesis | Human Diseases | Cancer: overview |
| map05230 | 0.055 | 1.000 | **0.035** | Central carbon metabolism in cancer | Human Diseases | Cancer: overview |
| map04930 | 0.055 | 1.000 | **0.035** | Type II diabetes mellitus | Human Diseases | Endocrine and metabolic disease |
| map05165 | 0.055 | 1.000 | **0.035** | Human papillomavirus infection | Human Diseases | Infectious disease: viral |
| map00280 | 1.000 | 1.000 | **0.035** | Valine, leucine and isoleucine degradation | Metabolism | Amino acid metabolism |
| map00310 | 0.055 | 1.000 | **0.035** | Lysine degradation | Metabolism | Amino acid metabolism |
| map00380 | 1.000 | 1.000 | **0.035** | Tryptophan metabolism | Metabolism | Amino acid metabolism |
| map01100 | **0.000** | 1.000 | **0.000** | Metabolic pathways | Metabolism | Global and overview maps |
| map01110 | 0.055 | 1.000 | **0.001** | Biosynthesis of secondary metabolites | Metabolism | Global and overview maps |
| map01120 | **0.000** | **0.006** | **0.001** | Microbial metabolism in diverse environments | Metabolism | Global and overview maps |
| map01130 | **0.001** | 1.000 | **0.001** | Biosynthesis of antibiotics | Metabolism | Global and overview maps |
| map01200 | **0.000** | **0.006** | **0.001** | Carbon metabolism | Metabolism | Global and overview maps |
| map01212 | 1.000 | 1.000 | **0.035** | Fatty acid metabolism | Metabolism | Global and overview maps |
| map01230 | **0.001** | 1.000 | **0.035** | Biosynthesis of amino acids | Metabolism | Global and overview maps |
| map00860 | 1.000 | 1.000 | **0.035** | Porphyrin and chlorophyll metabolism | Metabolism | Metabolism of cofactors and vitamins |
| map00410 | 1.000 | 1.000 | **0.035** | beta-Alanine metabolism | Metabolism | Metabolism of other amino acids |
| map00281 | 1.000 | 1.000 | **0.035** | Geraniol degradation | Metabolism | Metabolism of terpenoids and polyketides |
| map00903 | 1.000 | 1.000 | **0.035** | Limonene and pinene degradation | Metabolism | Metabolism of terpenoids and polyketides |
| map04922 | 0.055 | 1.000 | **0.035** | Glucagon signaling pathway | Organismal Systems | Endocrine system |
