# Supplementary material for: Roundup causes embryonic development failure and alters metabolic pathways and gut microbiota functionality in non-target species
Source: Microbiome. 2020 Dec 15;8:170. doi: 10.1186/s40168-020-00943-5 (PMC7780628; doi:10.1186/s40168-020-00943-5)
Supplement: Supplementary file 2 — Additional file 1. [file 40168_2020_943_MOESM1_ESM.zip › Suppa_etal_Additional File1 (appendix)_ESM.docx]

**Roundup causes embryonic development failure, alters metabolic pathways and gut microbiota functionality in non-target species**

Antonio Suppa^1,2^, Jouni Kvist^3^, Xiaojing Li^1^,Vignesh Dandhapani^1^, Hanan Almulla^1^, Camilla Avanzi^2^, Antoine Y. Tian^4^, Stephen Kissane^1^, Jiarui Zhou^1^, Alessio Perotti^5^, Hayley Mangelson^6^, Kyle Langford^6^, Valeria Rossi^2^, James B. Brown^7,8,9,#^ and Luisa Orsini^1,8,#^*

^1^Environmental Genomics Group, School of Biosciences, the University of Birmingham, Birmingham B15 2TT, UK

^2^ Department of Chemistry, Life Sciences and Environmental Sustainability University of Parma, Department of Life Sciences, Viale Usberti, 11/A Parma, Italy

^3^School of Biosciences, University of Birmingham, Birmingham B15 2TT, UK

^4^Computer Science, University of Birmingham, Birmingham, UK

^5^School of Biosciences, University of Birmingham, Edgbaston, Birmingham B15 2TT, UK

^6^Phase Genomics, Seattle, WA, USA

^7^Environmental Bioinformatics, Centre for Computational Biology, School of Biosciences, University of Birmingham Edgbaston, Birmingham, B15 2TT, UK

^8^Environmental Genomics and Systems Biology Division, Lawrence Berkeley National Laboratory, Berkeley, California 94720, USA.

^9^Statistics Department, University of California, Berkeley, Berkeley, CA, 94720 USA, Preminon LLC, Rodeo, CA, 94572 USA

#Shared senior authorship

*Corresponding author

***D. magna* reference microbiota, dynamics and origin**

The dynamics of the *Daphnia* microbiome, as well as its origin, are not well understood. Our first task was to create a reference gut metagenome. Following this task, we determined the size and composition of the gut microbiota from gut colonization (48h after birth) to last instar (144h after birth). We then established whether the gut microbiota was genetically determined. These tasks informed the core experiment of this study, in which we analyzed the impact of Glyphosate and Roundup on the established gut communities.

To create a reference gut metagenome, we used adult clones of a single *Daphnia* genotype (LRV0_1), for which the genome has been sequenced, using the Hi-C technology ^1^. Chromatin conformation capture data was generated using a Phase Genomics (Seattle, WA) Proximo Hi-C 2.0 Kit, which is a commercially available version of the Hi-C protocol ^1^. Following the manufacturer's instructions for the kit, intact cells from two samples were crosslinked using a formaldehyde solution, simultaneously digested using the Sau3AI and MlucI restriction enzymes, and proximity ligated with biotinylated nucleotides to create chimeric molecules composed of fragments from different regions of the genome that were physically proximal in vivo. Molecules were pulled down with streptavidin beads and processed into an Illumina-compatible sequencing library. Sequencing was performed on an Illumina HiSeq 4000, generating a total of 235,051,495 PE read pairs.

Shotgun reads were filtered and trimmed for quality using fastp ^2^, normalized using bbtools ^3^ and assembled with MEGAHIT ^4,5^ using default options. Hi-C reads were then aligned to the *Daphnia* assembly following the Hi-C kit manufacturer's recommendations to mask eukaryote sequences (https://phasegenomics.github.io/2019/09/19/hic-alignment-and-qc.html). Briefly, reads were aligned using BWA-MEM ^6^ with the -5SP and -t 8 options specified, and using the 2.4 *D. magna* draft assembly (GenBank LRGB00000000) (Dmagna-xinb3-Genome-Largethan1Kb-masurca.fasta) for host-masking. SAMBLASTER ^7^ was used to flag PCR duplicates, which were later excluded from the analysis. Alignments were then filtered with samtools ^8^ using the -F 2304 filtering flag to remove non-primary and secondary alignments. Metagenome deconvolution was performed with ProxiMeta ^9,10^. The taxonomic classification of the gut bacterial genomes generated with the Hi-C technology was derived with MASH ^11,12^. For strains with unknown classification, we used *pplacer* to identify the closest taxonomic classification at Family level ^13^. Completeness and novelty scores of the sequenced genomes were assessed using CheckM ^14^.

To determine the dynamics and composition of the microbiota during gut colonization, we applied metabarcoding to clonal replicates of the genotype LRV3.5_15, used in the core experiment. We quantified size and dynamics of the gut microbiome at different life stages from gut colonization (48h after birth) to last instar (144h after birth). To quantify the potential impact of environmental contaminants on the microbiota, we quantified the microbiota dynamics and composition in non-exposed clonal replicates as well as in clonal replicates exposed to environmentally relevant concentrations of two xenobionts, a commonly used carbamate insecticide (4µg/L; of Carbaryl, Pestanatal) and the herbicide Glyphosate (1mg/L). We used xenobionts with different mode of actions to achieve a comprehensive understanding of the *Daphnia* gut microbiota dynamics responding to diverse perturbations.

To assess whether the gut microbiome was genetically determined, we exposed clonal replicates of a recipient genotype (LRV3.5_15) to a donor microbiome (LRV 13.5_1) for 48h following treatment with antibiotics (20 mg/L final concentration of Tetracycline, Streptomycin and Ampicillin) in sterile medium for 12h. The donor and recipient microbiota were characterized before the experiment to select genotypes with distinct microbiota. After antibiotic treatment, the clonal replicates were exposed for 48 h to macerated guts from the donor genotype; the microbiota composition of the recipient genotype was quantified in non-exposed clones (control) as well as in clones exposed to Glyphosate (1 mg/L) and Roundup (Monsanto) (1 mg/L), which were relevant to the core experiment, to quantify the effect of early exposure on microbiota establishment. This experiment was conducted in non-sterile borehole medium as well as in synthetic bacteria-free COMBO medium ^15^.

**Results and discussion**

We created a reference metagenome for *D. magna* using the Hi-C technology ^1^. A total of twenty-three bacterial genomes were identified to form the gut microbiota of a healthy *Daphnia* (NCBI PRJNA606209; Table A1; Fig. A1). The genomes with a novelty score above 96% include taxa from the family *Burkholderiales* (Bacterioplankton), *Cytophagales* (unicellular gliding gut bacteria), *Bacteroidetes* (symbionts involved in the degradation of proteins and complex sugar polymers) and *Flavobacteriaceae* (aerobic freshwater bacteria). The abundance of the 23 genomes ranged between 13.5 % for taxa showing similarity to Bacterioplankton and 1.7% for other unknown bacteria (Table A1). *Burkholderiales* are gram-negative Proteobacteria belonging to freshwater Bacterioplankton; some strains are capable of nitrogen fixation ^16^. *Cytophagales* are gram-negative bacteria able to degrade biomacromolecules like proteins, chitin, pectin, agar, starch, or cellulose ^17^.*Bacteroidetes* are gram-negative bacteria common both in environmental matrices and in the gut of animals; they have been shown to carry enzymes for the degradation of high molecular weight organic matter i.e. proteins and carbohydrates ^18^. *Flavobacteriaceae* are the largest family in the phylum Bacteroidetes, which contains more than 90 genera; they are aerobic bacteria found in environmental matrices, and occasionally associated with animals. Genome analyses suggest that most members of the family are used as a protein secretion system ^19^.

We established the origin of the *Daphnia* gut microbiota by transplanting the gut of a donor genotype into a recipient genotype, which had distinct gut communities (Fig. A2). Replicate clones of the recipient genotype after transplant with the donor microbiome were more similar to the untreated recipient clonal replicates than to the donor (Fig. A2; Table A2, G). Because the antibiotic treatment efficiently cleansed the resident gut community (Fig. A3), and the non-sterile medium (borehole water) showed a bacterial composition significantly different from the gut community (Fig. A2), the similarity between the recipient genotype after transplant and the untreated recipient genotype can only be explained by the reestablishment of the same microbiota. This result contrasts with a recent study in which the authors conclude that the microbiota is horizontally transmitted from the environment ^20^. There are two explanations for the divergent results. Firstly, Macke *et al*. characterized the impact of the gut transplant indirectly, by assessing fitness responses rather than quantifying the gut community before and after transplant. We quantified the gut community at high depth of sequencing (>100,000 reads per sample and biological replicate) in the donor, the recipient and the transplanted genotype in sterile and non-sterile medium. Moreover, we quantified the microbial composition of the non-sterile medium, which was distinct from the gut community. Macke *et al.* only used sterile medium, making it challenging to determine the source of the microbiota. Secondly, Macke *et al*. created germ-free lines by isolating parthenogenetic eggs from gravid *Daphnia* and placing them in sterile medium. However, if the microbiota is vertically transmitted, transferring developed embryos to sterile medium after their development in the brood pouch will not lead to germ-free juveniles. If the microbiota is horizontally transmitted embryos in sterile medium should not develop a microbiota. To obtain germ-free *Daphnia*, we exposed biological replicates of a single genotype to a powerful cocktail of antibiotics, visualizing the content of the guts before and after this treatment to verify that the experimental animals’ microbiota was efficiently cleansed (Fig. A3), even though a complete removal was not possible because it causes the host’s death.

The number of bacterial taxa identified in our study is higher than previous studies in which up to 7 taxa were identified ^20,21^.This is expected because the *Daphnia* clone used in these studies was exposed to a single antibiotic agent, which may have targeted specific bacteria, ^21^ or different diets which may select for certain bacteria families ^22^. Furthermore, we are the first to fully characterize the reference metagenome of *D. magna* using an optimal tool for metagenome deconvolution, whereas previous studies rely on metabarcoding with 16S, which underestimate rare bacteria and it is unable to correctly identify novel taxa. We identify the same bacterial families with metabarcoding and metagenomics, providing independent evidence of the gut bacterial composition.

After establishing that the gut microbiota is strongly genetically determined, we quantified the impact of Glyphosate and Roundup on the re-establishment success and the composition of the gut microbiota post donor transplant. Glyphosate exposure significantly reduced the reestablishment of *Moraxellaceae* and *Caulobacteracea* (Table A3). Additionally, two families (*Burkholderiaceae, Flavobacteriaceae*) and five genera (*Perlucidibaca, Acidovorax, Curvibacter, Flavobacterium, Chryseobacterium*), largely cleansed by antibiotics treatment (Table A3; Co+A) prior to donor faecal microbiota transplant never recovered in presence of Roundup and Glyphosate (Table A3). Conversely, the family *Weeksellaceae* and the genus *Chryseobacterium* became proportionally more abundant (Table A3).

We determined the size and composition of the gut microbiota from gut colonization (48h after birth) to last instar (144h after birth) in control condition and after exposure to xenobiotic agents commonly found in human-impacted environments [Glyphosate and a carbamate insecticide (Carbaryl)]. Glyphosate is expected to have an impact on the gut community via the EPSPS enzyme ^23^. Carbamates are widely used in agriculture because they interfere with the insect neurotransmitter acetylcholinesterase, reducing their impact on crop ^24^. Although Carbamates should not target bacteria, they have been shown to perturb gut communities ^25,26^. The microbiota richness showed a clear pattern over time; diversity peaked at day four and stabilized after day five in both control animals and animals treated with xenobionts (Fig. A4). Hence, the xenobiotic agents did not alter the gut colonization process. However, exposures during early development to xenobiotic agents affected the composition of the gut microbiota and interfered with the normal colonization of bacterial species, leading to significantly different established gut communities (Fig. A4B; Table A3). Species richness of both common and rare gut bacteria species was significantly reduced by the xenobiotic agents (Fig. A4C; Table A3). As only active ingredients were used in this experiment, the alteration of the gut microbiota can only be explained by the direct impact of these ingredients on the bacterial community.

**References**

1 Lieberman-Aiden, E. *et al.* Comprehensive mapping of long-range interactions reveals folding principles of the human genome. *Science* **326**, 289-293, doi:10.1126/science.1181369 (2009).

2 Chen, S., Zhou, Y., Chen, Y. & Gu, J. fastp: an ultra-fast all-in-one FASTQ preprocessor. *Bioinformatics* **34**, i884-i890, doi:10.1093/bioinformatics/bty560 (2018).

3 Software, O. S. *BBMap short read aligner, and other bioinformatic tools.*

4 Li, D., Liu, C. M., Luo, R., Sadakane, K. & Lam, T. W. MEGAHIT: an ultra-fast single-node solution for large and complex metagenomics assembly via succinct de Bruijn graph. *Bioinformatics* **31**, 1674-1676, doi:10.1093/bioinformatics/btv033 (2015).

5 Li, D. *et al.* MEGAHIT v1.0: A fast and scalable metagenome assembler driven by advanced methodologies and community practices. *Methods* **102**, 3-11, doi:10.1016/j.ymeth.2016.02.020 (2016).

6 Li, H. & Durbin, R. Fast and accurate long-read alignment with Burrows-Wheeler transform. *Bioinformatics* **26**, 589-595, doi:10.1093/bioinformatics/btp698 (2010).

7 Faust, G. G. & Hall, I. M. SAMBLASTER: fast duplicate marking and structural variant read extraction. *Bioinformatics* **30**, 2503-2505, doi:10.1093/bioinformatics/btu314 (2014).

8 Li, H. *et al.* The Sequence Alignment/Map format and SAMtools. *Bioinformatics* **25**, 2078-2079, doi:10.1093/bioinformatics/btp352 (2009).

9 Press, M. O. *et al.* Hi-C deconvolution of a human gut microbiome yields high-quality draft genomes and reveals plasmid-genome interactions. *bioRxiv*, 198713 (2017).

10 Stewart, R. D. *et al.* Assembly of 913 microbial genomes from metagenomic sequencing of the cow rumen. *Nat Commun* **9**, 870, doi:10.1038/s41467-018-03317-6 (2018).

11 Ondov, B. D. *et al.* Mash: fast genome and metagenome distance estimation using MinHash. *Genome Biol* **17**, 132, doi:10.1186/s13059-016-0997-x (2016).

12 Ondov, B. D. *et al.* Mash Screen: high-throughput sequence containment estimation for genome discovery. *Genome Biol* **20**, 232, doi:10.1186/s13059-019-1841-x (2019).

13 Matsen, F. A., Kodner, R. B. & Armbrust, E. V. pplacer: linear time maximum-likelihood and Bayesian phylogenetic placement of sequences onto a fixed reference tree. *BMC Bioinformatics* **11**, 538, doi:10.1186/1471-2105-11-538 (2010).

14 Parks, D. H., Imelfort, M., Skennerton, C. T., Hugenholtz, P. & Tyson, G. W. CheckM: assessing the quality of microbial genomes recovered from isolates, single cells, and metagenomes. *Genome Res* **25**, 1043-1055, doi:10.1101/gr.186072.114 (2015).

15 Kilham, S. S., Kreeger, D. A., Lynn, S. G., Goulden, C. E. & Herrera, L. COMBO: a defined freshwater culture medium for algae and zooplankton. *Hydrobiologia* **377**, 147-159 (1998).

16 Woese, C. R., Kandler, O. & Wheelis, M. L. Towards a natural system of organisms: Proposal for the domains Archaea, Bacteria, and Eucarya. *Proceeding of the Natural Academy of Science USA* **87**, 4576-4579 (1990).

17 Reichenbach, H. *The Order Cytophagales*. (Springer, 2006).

18 Thomas, F., Hehemann, J. H., Rebuffet, E., Czjzek, M. & Michel, G. Environmental and gut bacteroidetes: the food connection. *Front Microbiol* **2**, 93, doi:10.3389/fmicb.2011.00093 (2011).

19 McBride, M. J. *The Family Flavobacteriaceae*. (Springer, 2014).

20 Macke, E., Callens, M., De Meester, L. & Decaestecker, E. Host-genotype dependent gut microbiota drives zooplankton tolerance to toxic cyanobacteria. *Nat Commun* **8**, 1608, doi:10.1038/s41467-017-01714-x (2017).

21 Callens, M., Watanabe, H., Kato, Y., Miura, J. & Decaestecker, E. Microbiota inoculum composition affects holobiont assembly and host growth in Daphnia. *Microbiome* **6**, 56, doi:10.1186/s40168-018-0444-1 (2018).

22 Callens, M. *et al.* Food availability affects the strength of mutualistic host–microbiota interactions in Daphnia magna. *International Society for Microbial Ecology* **10**, 911-920 (2016).

23 Pollegioni, L., Schonbrunn, E. & Siehl, D. Molecular basis of glyphosate resistance - different approaches through protein engineering. *Febs J* **278**, 2753-2766, doi:10.1111/j.1742-4658.2011.08214.x (2011).

24 Assis, C. R. D. *et al.* Erythrocyte acetylcholinesterase as biomarker of pesticide exposure: new and forgotten insights. *Environ Sci Pollut R* **25**, 18364-18376 (2018).

25 Gao, B., Chi, L., Tu, P., Gao, N. & Lu, K. The Carbamate Aldicarb Altered the Gut Microbiome, Metabolome, and Lipidome of C57BL/6J Mice. *Chem Res Toxicol* **32**, 67-79, doi:10.1021/acs.chemrestox.8b00179 (2019).

26 Wu, S., Jin, C., Wang, Y., Fu, Z. & Jin, Y. Exposure to the fungicide propamocarb causes gut microbiota dysbiosis and metabolic disorder in mice. *Environ Pollut* **237**, 775-783, doi:10.1016/j.envpol.2017.10.129 (2018).

27 Chao, A. & Chiu, C.-H. Species Richness: Estimation and Comparison. *Encyclopedia of Statistical Sciences* **Statistics Reference Online**, 1-26 (2016).

**Figure A1. Reference metagenome**. Novelty and abundance of the gut microbes assembled using the Proximeta metagenome deconvolution approach ^10^.

**
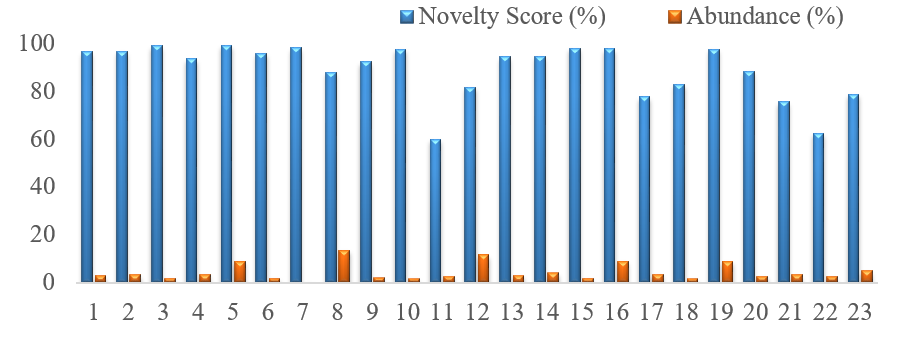
**

**Figure A2. Gut microbiota transplant**. Gut microbiota OTU profile based on the 10 most abundant bacterial families for: 1) the donor genotype (LRV 13.5_1) gown in sterile medium (SM); 2) the recipient genotype (LRV 3.5_15) grown in sterile (SM) and non-sterile medium (NSM); 3) the recipient genotype after exposure to macerated guts of the donor genotype post antibiotic treatment (SMT); 4) the microbial composition of the non-sterile medium (borehole water).

**
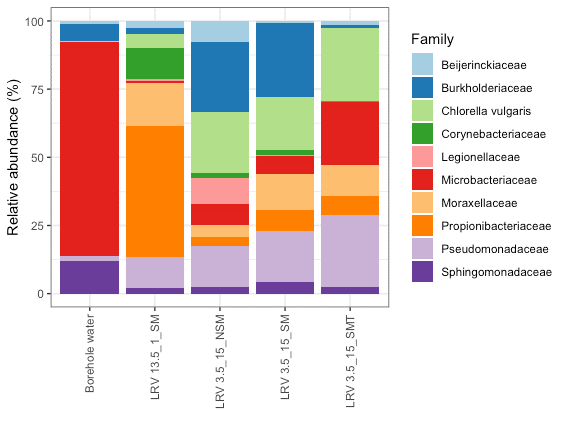
**

**Figure A3. Antibiotic treatment for gut microbiota cleansing**. Adult *Daphnia* exposed to different concentrations of an antibiotic cocktail including Tetracycline, Streptomycin and Ampicillin for 72h. A) Adult *Daphnia* not exposed to antibiotics; and adult *Daphnia* exposed to B) 5 mg/L; C) 10 mg/L; D) 15 mg/L; E) 20 mg/L; and F) 25 mg/L of antibiotics. The arrows point to the guts.


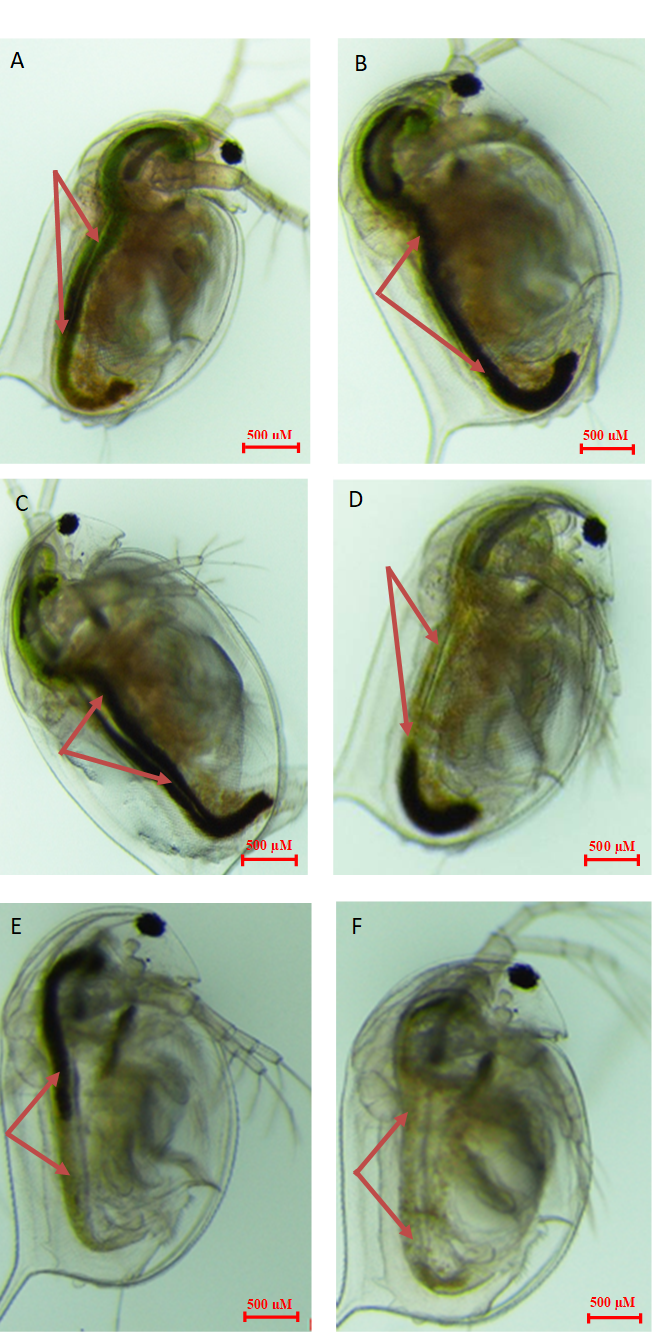


**Figure A4. Establishment of gut communities.** Mean and standard deviations for overall species richness (Chao 1 ^27^) (A) and proportion of rare OTUs (B) are shown from gut colonization (48h after birth, Day 3) to last instar (144h after birth; Day 6) in control conditions and after exposure to the insecticide Carbaryl (4µg/L) and the weed killer Glyphosate (1mg/L). Changes in the composition of the gut microbiota from gut colonization (Day1) to established communities (Day 6) averaged across biological replicates is shown (C) for the 10 most abundant bacteria families in control conditions (Co) and after exposure to Carbaryl (Ca) and Glyphosate (Gly). Supporting statistics are in Table A3.

**
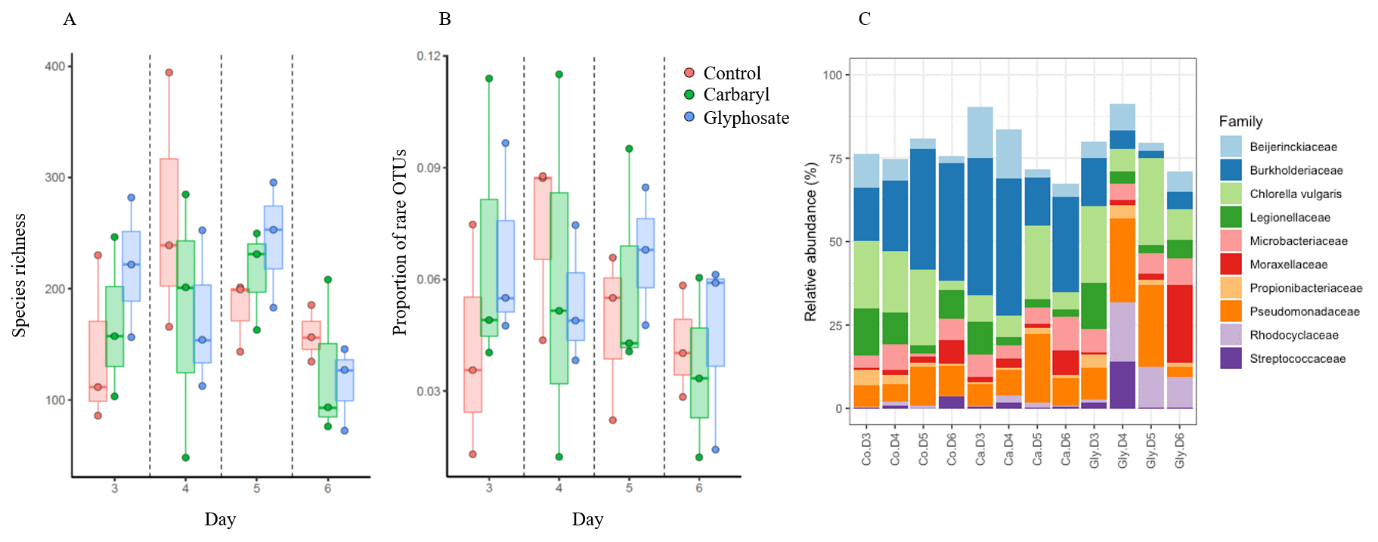
**

**Table A1. ProxiMeta assembly.** List of novel bacterial genomes assembled using the Hi-C phasegenomics technology. Completeness, novelty score, abundance, N50 size, estimated genome size, total number of contigs per genome and GC content are shown. The listed family names are the top hits based on sequence similarity.

| Bacteria sp - closest hit | Complete (%) | Novelty Score (%) | Abundance (%) | N50 | Genome Size (Mb) | Number Contigs | GC (%) |
| --- | --- | --- | --- | --- | --- | --- | --- |
| *Burkholderiales (proteobacteria, Bacterioplankton)** | 93.40 | 96.62 | 2.98 | 44,662 | 4,962,466 | 139 | 66.46 |
| *Cytophagales (Cytophaga, unicellular gliding bacteria)** | 86.14 | 96.79 | 3.41 | 35,083 | 4,383,112 | 215 | 36.21 |
| *Cytophagales (Cytophaga, unicellular gliding bacteria)** | 80.44 | 99.01 | 1.87 | 18,925 | 5,206,054 | 326 | 35.69 |
| *Burkholderiales (proteobacteria, Bacterioplankton)* | 78.82 | 93.56 | 3.43 | 3,834 | 3,219,835 | 999 | 60.70 |
| *Bacteroidetes (symbiont, degradation of proteins and complex sugar polymers)* | 57.97 | 99.31 | 8.94 | 56,159 | 2,272,343 | 45 | 33.54 |
| *Bacteria* | 48.40 | 95.93 | 1.77 | 4,604 | 1,113,425 | 312 | 48.57 |
| *Deltaproteobacteria (Proteobacteria, sulfate-reducing bacteria)* | 42.65 | 98.14 | - | 1,597 | 2,557,958 | 1613 | 65.20 |
| *Burkholderiales (proteobacteria, Bacterioplankton)* | 41.14 | 87.95 | 13.46 | 14,858 | 2,147,399 | 197 | 59.67 |
| *Betaproteobacteria (Proteobacteria, eutrophs)* | 35.29 | 92.30 | 1.99 | 1,707 | 2,773,488 | 1605 | 49.47 |
| *Flavobacteriaceae(Proteobacteria, aerobic freshwater)* | 33.58 | 97.64 | 1.75 | 3,436 | 2,228,515 | 855 | 39.25 |
| *Bacteria* | 31.63 | 59.93 | 2.78 | 2,185 | 2,491,131 | 1199 | 44.97 |
| *Bacteria* | 30.30 | 81.62 | 11.74 | 7,002 | 1,566,997 | 254 | 60.51 |
| *Bacteria* | 28.12 | 94.43 | 3.18 | 2,743 | 2,126,138 | 871 | 57.57 |
| *Burkholderiales(proteobacteria, Bacterioplankton)* | 21.98 | 94.65 | 4.04 | 1,759 | 1,870,018 | 1052 | 47.54 |
| *Bacteria* | 19.64 | 97.98 | 1.88 | 22,761 | 151,069 | 6 | 36.95 |
| *Bacteria* | 18.97 | 97.91 | 8.92 | 27,076 | 740,640 | 40 | 32.98 |
| *Burkholderiales (proteobacteria, Bacterioplankton)* | 18.06 | 77.77 | 3.46 | 1,601 | 780,575 | 453 | 59.27 |
| *Bacteria* | 17.54 | 82.97 | 1.68 | 2,044 | 2,913,505 | 1478 | 58.32 |
| *Unknown* | 16.67 | 97.62 | 9.00 | 4,185 | 675,116 | 214 | 33.23 |
| *Bacteria* | 13.27 | 88.48 | 2.71 | 1,918 | 439,304 | 215 | 58.83 |
| *Bacteria* | 11.83 | 75.57 | 3.40 | 2,361 | 560,087 | 252 | 60.24 |
| *Bacteria* | 7.21 | 62.47 | 2.57 | 1,522 | 976,105 | 607 | 58.02 |
| *Bacteria* | 1.72 | 78.69 | 5.04 | 3,118 | 2,068,396 | 787 | 40.92 |

**Table A2. Transplant experiment.** Permutational Multivariate Analysis of Variance using OTU Bray Curtis distance matrices testing for genotype differences, including the transinfected Genotype (G), Treatment (T) including Glyphosate and Roundup on antibiotic treated and untreated genotypes, and the interaction term between genotype and treatment (G x T). significant values are in bold.

|  | Df | R2 | Pr(>F) |
| --- | --- | --- | --- |
| Genotype (G) | 2 | 0.23 | **1E-04** |
| Treatment (T) | 2 | 0.08 | **0.02** |
| Antibiotics | 1 | 0.08 | **0.001** |
| G x T | 4 | 0.16 | **0.004** |

**Table A3. Core taxa altered by exposure to treatment post donor transplant.** Relative abundance of bacterial taxa measured in the recipient genotype LRV3.5_15 in control untreated conditions (Co), in germ-free replicates (Co+A), and in germ-free exposed replicates to Roundup (Rou) and Glyphosate (Gly). Changes in relative abundance, reported at Family and Genus level, are calculated for each day using Control as reference. Core taxa, taxa with abundance > than 1% across treatments and detected in at least 90% of the samples, are underlined. *P*-values are adjusted using the Benjamini-Hochberg method with a false discovery rate of 0.05. Significant values are in bold.

| **Phylum** | **Family** | **Co** | **Co+A** | **Gly** | **Rou** | **Genus** | | **Co** | **Co+A** | **Gly** | **Rou** |
| --- | --- | --- | --- | --- | --- | --- | --- | --- | --- | --- | --- |
| *Proteobacteria* | *Moraxellaceae* | 19.75% | 4.81% | **0.49%**** | 23.00% | *Acinetobacter* | | 10.40% | 4.79% | 0.49% | 22.97% |
|  |  |  |  |  |  | *Perlucidibaca* | | 9.30% | **0.00%**** | **0.00%**** | **0.00%**** |
|  | *Pseudomonadaceae* | 15.19% | 17.07% | 14.67% | 33.64% | *Pseudomonas* | | 15.19% | 17.07% | 14.67% | 33.64% |
|  | *Burkholderiaceae* | 30.59% | **0.79%***** | **1.15%***** | **2.14%***** | *Dechlorosoma* | | 0.09% | 0.15% | 2.07% | 0.14% |
|  |  |  |  |  |  | *Limnohabitans* | | 9.60% | 0.20%* | 0.59% | 0.71% |
|  |  |  |  |  |  | *Acidovorax* | | 4.42% | **0.02%**** | **0.00%***** | **0.06%*** |
|  |  |  |  |  |  | *Curvibacter* | | 9.84% | **0.00%**** | **0.00%**** | **0.00%**** |
|  | *Rhodocyclaceae* | 0.18% | 0.15% | 2.07% | 0.14% |  | |  |  |  |  |
|  | *Beijerinckiaceae* | 0.58% | 0.33% | 2.72% | 1.59% | *Methylobacterium* | | 0.41% | 0.24% | 2.15% | 1.17% |
|  | *Xanthobacteraceae* | 0.16% | **2.46%*** | 0.63% | 1.20% | *Bradyrhizobium* | | 0.00% | **1.80%*** | 0.32% | 0.46% |
|  | *Sphingomonadaceae* | 4.49% | **0.52%**** | 4.21% | 3.73% | *Sphingobium* | | 4.03% | **0.33%*** | 4.12% | 2.66% |
|  | *Caulobacteraceae* | 1.66% | 0.60% | **0.00%*** | 2.66% | *Brevundimonas* | | 1.66% | 0.59% | 0.00% | 2.62% |
| *Actinobacteria* | *Propionibacteriaceae* | 1.44% | 20.45% | 7.21% | 1.69% | *Cutibacterium* | | 1.42% | 20.44% | 7.17% | 1.67% |
|  | *Microbacteriaceae* | 0.81% | 18.03% | 27.91% | 12.52% | *Galbitalea* | | 0.28% | 16.66% | 20.23% | 3.87% |
|  |  |  |  |  |  | *Microbacterium* | | 0.18% | 1.04% | 7.07% | 8.29% |
|  | *Corynebacteriaceae* | 0.12% | **2.26%*** | 0.91% | 0.30% | *Corynebacterium 1* | | 0.12% | 2.21% | 0.91% | 0.28% |
|  | *Nocardiaceae* | 0.75% | 6.86% | 23.46% | 12.15% | *Rhodococcus* | | 0.46% | 6.44% | 21.27% | 10.99% |
|  |  |  |  |  |  | *Gordonia* | | 0.28% | 0.41% | 2.14% | 1.16% |
| *Firmicutes* | *Streptococcaceae* | 0.00% | 1.65%* | 0.13% | 0.01% | *Streptococcus* | | 0.00% | **1.21%*** | 0.12% | 0.01% |
|  | *Staphylococcaceae* | 0.07% | 2.28%* | 0.99% | 0.26% | *Staphylococcus* | | 0.06% | 2.27% | 0.96% | 0.26% |
| *Bacteroidetes* | *Flavobacteriaceae* | 20.90% | **0.00%***** | **0.00%***** | **0.00%***** | *Flavobacterium* | | 20.90% | **0.00%**** | **0.00%**** | **0.00%**** |
|  | *Weeksellaceae* | 0.00% | **2.96%***** | **0.12%***** | **1.64%***** | *Chryseobacterium* | | 0.00% | **2.96%***** | **0.12%***** | **1.44%***** |
| *Dependentiae* | *Vermiphilaceae* | 0.00% | 0.00% | 2.56% | 0.01% |  | |  |  |  |  |
| **sum** |  | 96.68% | 78.25% | 86.56% | 95.02% |  | 88.64% | | 75.88% | 84.28% | 90.96% |
